# Selection of immunoglobulin elbow region mutations impacts interdomain conformational flexibility in HIV-1 broadly neutralizing antibodies

**DOI:** 10.1038/s41467-019-08415-7

**Published:** 2019-02-08

**Authors:** Rory Henderson, Brian E. Watts, Hieu N. Ergin, Kara Anasti, Robert Parks, Shi-Mao Xia, Ashley Trama, Hua-Xin Liao, Kevin O. Saunders, Mattia Bonsignori, Kevin Wiehe, Barton F. Haynes, S. Munir Alam

**Affiliations:** 1 Department of Medicine, Duke University School of Medicine, Durham, NC 27710 USA; 2 Department of Pathology, Duke University School of Medicine, Durham, NC 27710 USA; 3 Present Address: College of Life Science and Technology, Jinan University, Guangzhou, 510632 China

## Abstract

Somatic mutations within antibody variable and framework regions (FWR) can alter thermostability and structural flexibility, but their impact on functional potency is unclear. Here we study thermostability and use molecular dynamics (MD) simulations to assess the role of FWR mutations during maturation of HIV-1 broadly neutralizing antibodies (bnAbs). The tested bnAbs show lower thermostability than their unmutated ancestor antibodies. FWR mutations in the Fab elbow region are frequently observed in HIV-1 bnAbs and MD simulations show that such FWR mutations alter interdomain flexibility in two HIV-1 bnAbs. In a CD4-binding site lineage, reversion mutations result in a loss of neutralization potency in an early intermediate and affinity-matured bnAb against autologous and heterologous Tier-2 viruses, respectively. Elbow region reversion mutations in a glycan-V3 bnAb modestly reduces potency against an autologous virus isolate. Thus, selection of mutations in the Fab elbow region impacts interdomain conformational flexibility and paratope plasticity during bnAb development.

## Abstract (web summary)

Somatic mutations within antibody framework regions (FWR) can alter structural flexibility, but their role in maturation of broadly neutralizing antibodies (bnAbs) is unclear. Here the authors show how FWR mutations impact interdomain conformational flexibility and paratope plasticity during bnAb development.

## Introduction

Affinity maturation of antibodies involves mutations both within the antigen-binding site as well as in distal sites in the antibody framework regions (FWR)^1,2^. Various roles attributed to FWR residues include being neutral to scaffolding for antibody structural integrity, compensating for destabilizing CDR (complementarity determining region) mutations, and enhancing variable loop flexibility^2–5^. Affinity-enhancing mutations in residues that directly interact with antigen can be detrimental to antibody thermostability, and as such, germline-reverted mutants are thermodynamically less stable when compared to affinity-matured antibodies^5–8^. Since the destabilizing effect of affinity-enhancing mutations is overcome by concurrent selection of stabilizing mutations, affinity maturation has been viewed as a selection process that optimizes both antibody affinity and thermostability^5^. As observed in the evolution of enzymes, a function/stability trade-off occurs during antibody affinity maturation and therefore, co-selection of mutations in CDRs and FWRs are required to maintain a balance between antibody function and stability^5,9,10^.

The relative disposition of the F_v_ (Fab variable) to the C_H1_/C_L_ (constant heavy1/constant light), and the relative orientation of the heavy and light chain variable domains, V_H_ and V_L_, can be altered by FWR mutations^11,12^. The former describes a secondary region of flexibility termed the Fab elbow^11,12^, distinct from the well-known antibody hinge region between the Fab and F_c_, while the latter determines the geometry of the binding site^11–13^. The Fab elbow adds an additional spatial degree of conformational flexibility which is not necessarily fixed but may display dynamic flexibility, capable of shifting in the presence of ligand^4,14^. Thus, during affinity maturation, the selection of FWR mutations in the Fab elbow residues can be important for optimizing antibody conformational dynamics and adaptation to antigen structure^4,15,16^.

The role of FWR mutations and the molecular basis for their selection during affinity maturation of HIV-1 broadly neutralizing antibodies (bnAbs) is not clearly understood. Recent studies indicate that distal mutations acquired in exposed loops and the FWRs are not all neutral but rather can contribute to antigen binding or enhance neutralization potency through modification of structural stability and/or loop flexibility^8,17–20^. FWR mutations can be thermally destabilizing in a fully matured bnAb^8,21^ and improvement in neutralization potency can incur a cost to thermostability^22^. However, FWR mutations were reported to provide no functional advantage for weakly neutralizing HIV-1 antibodies, while being essential for bnAb neutralization^8^. For a CD4-bs bnAb, germline reversion of a FWR residue that afforded loop flexibility increased the thermostability (melting temperature, T_m_) and decreased the neutralization potency^8^, indicating that the bnAb development incurred a stability cost in gaining functional potency. These studies highlight the importance of FWR mutations for bnAb development and the need for understanding the role of specific FWR mutations during bnAb maturation. The relationship between thermostability and gain-of-function for each antibody in a bnAb lineage, including the inferred unmutated common ancestor (UCA) and intermediate antibodies, remains undefined. In the early stages of bnAb development, the selection of mutations that contribute to interdomain flexibility can be advantageous in overcoming the geometric hurdles presented by the HIV-1 Env trimeric arrangement, as well as shifting variable loop lengths and the associated glycan positions^23,24^. Thus, fine-tuning paratope and Fab structural flexibility together likely plays a major role in determining the ability of maturing antibodies to develop heterologous breadth. The above considerations raise the question of whether any key mutations selected early in bnAb maturation impact antibody conformational flexibility and thermostability and pave a path in which concurrent and subsequent affinity-enhancing mutations are selected without further detriment to antibody stability. Thus, we aimed to identify the mutational changes that are destabilizing and/or contribute to interdomain flexibility in HIV-1 bnAb lineages targeting different HIV-1 Env epitopes and to determine if there is an association between the selection of such mutations and recognition of heterologous antigenic sequences and subsequent bnAb development.

Here, we describe the role of specific Ig V_H_ mutations in optimizing antibody thermostability and interdomain conformational flexibility during HIV-1 bnAb development. We show that all major classes of bnAbs have lower thermostability than their corresponding inferred UCA antibodies. The thermal destabilization in two bnAb lineages—the CD4-bs CH103^24^ and the glycan-V3 DH270^25^—was first observed in an early intermediate and was maintained in the affinity-matured antibodies that developed neutralization potency and breadth. In the CH103 bnAb lineage, we identified heavy chain FWR residues that contribute to thermal destabilization and show that mutations in the Fab elbow region altered interdomain flexibility and were important for bnAb development. We report that Fab elbow region mutations are frequently observed in HIV-1 bnAbs and using long timescale molecular dynamics (MD) simulations, we describe the role of these mutations in enhancing interdomain flexibility in the two bnAb lineages studied. Furthermore, we show that the selection of destabilizing elbow region mutations was important for neutralization in the CH103 lineage, since reversion of destabilizing mutations in an early intermediate and the affinity-matured bnAb resulted in a loss of neutralization potency. In the glycan-V3 DH270 lineage, reversion of elbow mutations resulted in the loss of neutralization potency in a bnAb against more difficult-to-neutralize viruses. Our studies demonstrate that mutations that impact Fab interdomain flexibility are selected early in the bnAb evolutionary path and are important for bnAb development.

## Results

### HIV-1 bnAbs are less thermostable than their UCAs

**Fig. 1:**
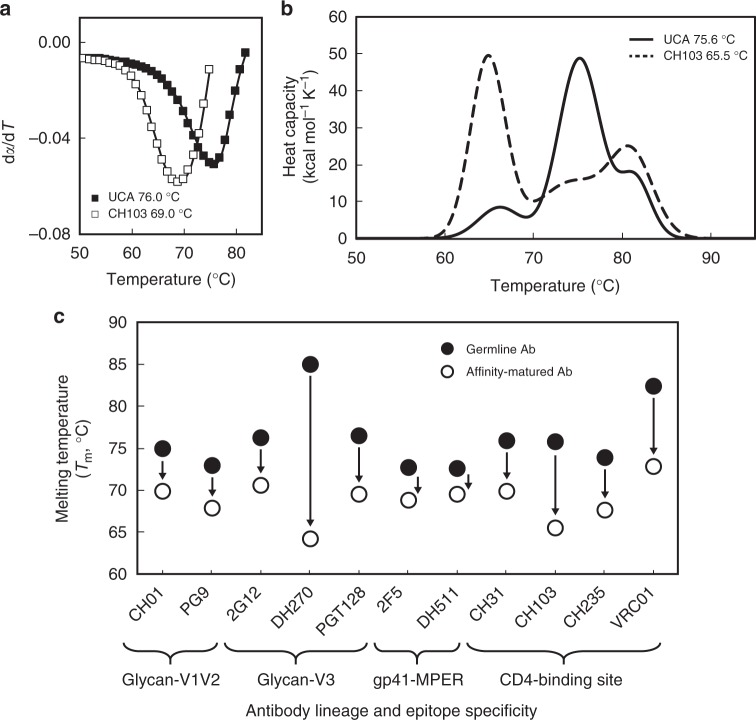
Thermal destabilization in bnAb affinity maturation. **a** Representative CD fraction folded derivative plots indicating the observed melting temperature (T_m_) for CH103 and its corresponding UCA. **b** Representative DSC model sum denaturation profiles for both CH103 and its corresponding UCA demonstrating destabilization of the Fab domain (largest amplitude peak) that is consistent with the CD analysis. The onset of Ab denaturation remains the same (~60 °C) for both the germline and mature Abs. **c** Thermal denaturation of affinity-matured HIV-1 bnAbs (open circle) and their corresponding germline or germline-proximal mAbs (closed circle) was performed by circular dichroism (CD) and/or differential scanning calorimetry (DSC) for Ab pairs of indicated epitope specificity. Data are plotted as the mean and standard deviation from a minimum of two replicate measurements. Error bars are smaller than the data markers at this scale. Data shown for CH01, PG9, and CH31 Ab pairs are the result of single measurements by CD with replicate measurements by DSC reported in Supplementary Table 1

To determine whether affinity maturation affected the stability of each of the major classes of HIV-1 bnAbs, we measured the melting temperatures (T_m_) of bnAbs and their inferred UCA antibodies by performing thermal denaturation by circular dichroism (CD) and differential scanning calorimetry (DSC) (Fig. 1). Each affinity-matured antibody displayed lower T_m_ than their corresponding germline or germline-proximal intermediate mAb, with the largest relative differences (>5 °C) observed for the CD4-bs and glycan-V3 bnAbs (Fig. 1c; Supplementary Table 1). Most notably, the glycan-V3 bnAb DH270 exhibited a ~20 °C reduction in T_m_ compared to its UCA. These data suggest that thermal destabilization during affinity maturation is observed in every major class of HIV-1 bnAb specificity.

### FWR mutations impact thermostability in the CH103 lineage

**Fig. 2:**
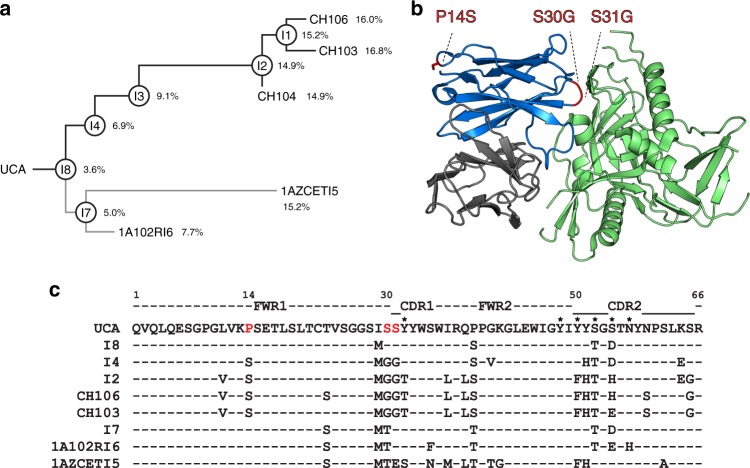
Heavy chain FWR1 mutations in the CH103 bnAb lineage. **a** The mature CH103 bnAbs were isolated from an HIV-1 infected African patient^24^. The unmutated common ancestor (UCA) and intermediate antibodies (Is) were inferred and each mAb was produced as described previously^24^. The % V_H_ mutations are indicated on the phylogram. **b** The location of the mutations (red) P14S (FWR1), S30G and S31G (HCDR1 proximal) that are selected in I4 are shown for the CH103 bnAb heavy chain (blue) in complex with gp120 (green). The CH103 light chain is shown in gray. The CH103-gp120 bound structure was originally solved in complex with only the outer domain of gp120^24^. For visualizing the location of the CH103 mutations in the context of a more fully resolved gp120, the CH103 complex structure was superimposed onto a gp120 core that included the inner domain (PDB: 4RQS). A portion of the V1V2 loop in the gp120 has been removed for clarity. **c** V_H_ sequence (partial) alignment of UCA, intermediates (I8, I4, I2, I7), mature CH103 bnAbs (CH106, CH103), and lower branch mAbs (1A102RI6, 1AZCETI5) depict the mutations acquired during affinity maturation. Mutations of ^14^P to S and ^30^SS to GG (shown in red) in the FWR1 and HCDR1 proximal regions, respectively, were first observed in the intermediate I4 and maintained in each of the later evolving intermediates leading up to the mature bnAb CH103. Interestingly, these mutations were not observed in the lower branch antibodies (I7, 1A102RI6, 1AZCETI5) and binding to heterologous Env (B.63521) was orders of magnitude weaker compared to the mAbs of the CH103 bnAb branch (Supplementary Table 5). Asterisks mark residues that make direct contact with antigen

We previously described the evolution of the CD4-bs CH103 bnAb lineage, which included the inferred UCA, and several inferred intermediates, that preceded the affinity-matured bnAbs with V_H_ mutation frequencies of 14.9–16.8% (Fig. 2a)^24,26^. To identify mutations that might have contributed to thermal destabilization of this bnAb, we compared the V_H_ sequences of the CH103 lineage antibodies and generated three CH103 UCA mutants (Fig. 2). We produced UCA mutant antibodies with single mutations in the heavy chain FWR1 at position 14 (UCA-P14S) and HCDR1 proximal position 30 (UCA-S30G). An additional mutation was introduced at position 31 of the UCA HCDR1 to generate the double mutant UCA-S30G/S31G. All three of the above mutations were selected in the germline-proximal intermediate I8 to I4 transition and were retained in each of the later intermediates and the mature antibodies in the upper branch of the CH103 lineage tree (Fig. 2). The positioning of the three substituted residues on a gp120-liganded model of CH103, following superposition of gp120 to the resolved CH103-bound state with gp120 core^24^, showed the remote location of the FWR1 P14S residue when compared to the closer proximity of HCDR1 proximal residues (S30G/S31G) to the antibody-antigen interface (Fig. 2b). Structural analysis of the UCA versus the CH103 bnAb revealed that the P14S mutation alters a loop between strands A and B while the CDR1 proximal S30G and S31G mutations increase loop and CDR1 flexibility respectively^24^. The serine residues (S30, S31) are adjacent to two tyrosine residues (Y32, Y33) in HCDR1, and the mutation of the serine residues (S30G, S31G) makes up the fully evolved MGGT motif, which was first complete in I2 and maintained in the bnAbs (Fig. 2c). Only the mutated HCDR1 Y33T residue showed contact with gp120 D368 when liganded to CH103^24^, and none of the three selected residues in the UCA mutant design (P14S, S30G, S31G) made any direct contact with gp120.

The CH103 UCA mutants had T_m_ values that were lower than the wild-type UCA, with the single mutants (UCA-P14S, UCA-S30G) each reduced by 1 °C and the double mutant (UCA-S30G/S31G) reduced by 1.8 °C (Table 1; Supplementary Table 2). These data suggest that FWR mutations and those that affect loop flexibility can impact thermostability. Furthermore, the binding affinities and rate constants (association/dissociation) of the UCA and each of the UCA mutants to the autologous CH505TF gp120, as well as to CH505TF gp140 SOSIP trimers, were similar, and neither the UCA nor the UCA mutants bound to the heterologous Env B.63521 gp120 (Table 2; Supplementary Table 3). Thus, the selected UCA V_H_ mutations (FWR1/CDR1 proximal) (Fig. 2), when introduced in the UCA, did not have any long-range configurational effect on the antibody’s binding site that provided any detectable affinity improvement beyond impacting thermal stability. Since each of the mutations were retained in the later intermediates and the isolated mature CH103 bnAbs, the destabilizing mutations, once selected, were fixed in the lineage. Therefore, we predicted that the thermal destabilization occurred in one of the earlier intermediate antibodies when the full sequence of mutations (^14^S…MGG^31^) was first acquired, as in intermediate I4 (Fig. 2).

**Table 1:** Effect of heavy chain mutations on thermostability in the CH103 lineage

mAb	T_m_ (°C)	ΔT_m_ (°C)
UCA	75.6 ± 0.3	--
UCA-P14S	74.63 ± 0.06	−1.0 ± 0.3
UCA-S30G	74.56 ± 0.06	−1.1 ± 0.3
UCA-S30G/S31G	73.8 ± 0.4	−1.8 ± 0.5
I4	64.60 ± 0.01	--
I4-S14P	67.01 ± 0.04	+2.41 ± 0.04
I4-S14P/G30S	68.42 ± 0.02	+3.82 ± 0.03
CH103	65.5 ± 0.1	--
CH103-S14P	66.41 ± 0.03	+0.9 ± 0.1
CH103-S14P/G30S	66.57 ± 0.05	+1.0 ± 0.1

Antibody thermal denaturation profiles were obtained and analyzed by DSC as described in Methods. Data are shown as the mean and standard deviation from a minimum of two replicate measurements. The ΔT_m_ is calculated for each mutant relative to wild-type with error propagated from the standard deviations

**Table 2:** Apparent affinity of the CH103 UCA mutant and lineage mAbs for transmitted founder (TF) and heterologous HIV-1 Env

mAb	C.CH505TF gp120 (*K*_d_ × 10^−9^ M)	C.CH505TF SOSIP.664.v4.1 (*K*_d_ × 10^−9^ M)	B.63521 D11 gp120 (*K*_d_ × 10^−9^ M)
UCA	430 ± 20	1,500 ± 300	NB
UCA-P14S	370 ± 30	1,700 ± 400	NB
UCA-S30G	400 ± 10	1,800 ± 100	NB
UCA-S30G/S31G	440 ± 20	800 ± 100	NB
I4	53 ± 5	60 ± 20	14,000 ± 3,000
I3	9.53 ± 0.06	ND	4200 ± 100
CH106	9.3 ± 0.8	5 ± 1	14.2 ± 0.9
CH103	40 ± 10	16 ± 3	1.1 ± 1.0

Apparent dissociation constants (*K*_d_) for each mAb were calculated from SPR kinetic analyses for autologous C.CH505TF gp120 and heterologous B.63521 D11 gp120 and from BLI kinetic analysis for autologous C.CH505TF.SOSIP.664.v4.1 as described in Methods. Data are shown as the mean and standard deviation from a minimum of two replicate measurements

*NB* no binding, *ND* not determined

### Thermal destabilization occurs early in the CH103 lineage

**Fig. 3:**
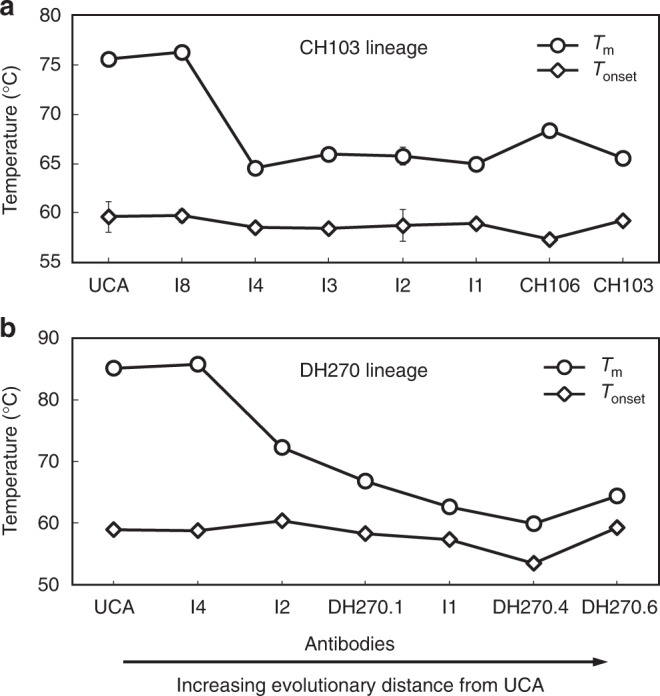
Thermostability in the CH103 and DH270 bnAb lineages. **a** DSC thermal denaturation analysis of the CH103 lineage mAbs reveals a marked decrease in Fab domain thermostability (T_m_) at the I8 to I4 transition (11.7 °C), after which, the thermal stability remains relatively constant throughout the remainder of the maturation process. The onset of whole antibody denaturation (T_onset_) remains constant throughout the lineage. **b** DSC thermal denaturation analysis of the DH270 lineage reveals a similar marked decrease in Fab domain thermostability at the earlier I4 to I2 transition (13.4 °C), followed by an additional 8.0 °C decrease in the mature bnAb DH270.6. Data are plotted as the mean and standard deviation from a minimum of two replicate measurements. Certain error bars are smaller than the data markers at this scale

To address how affinity maturation affected the thermostability of the intermediates, we measured T_m_ of the CH103 clonal lineage antibodies (Fig. 3a; Supplementary Figure 1, Table 4). T_m_ values for the UCA and the germline proximal intermediate I8 were similar, which was expected since I8 retained both the germline FWR1 P14 and HCDR1 proximal S30/31 residues. However, as predicted, I4 with the P14S/S30G/S31G mutations showed a marked decrease in T_m_ of 11.7 °C. Reversion of these mutations to produce single (I4-S14P) and double (I4-S14P/G30S) mutant antibodies resulted in increased T_m_ values relative to the wild-type I4, of 2.4 °C and 3.8 °C, respectively (Table 1; Supplementary Table 2). The above I4 mutations were fixed in the lineage and the lower T_m_ was subsequently maintained throughout the maturation pathway (Fig. 3a). T_onset_, the temperature at which whole antibody denaturation begins remained relatively constant throughout the maturation process (57–60 °C), and the DSC profiles indicated that the drop in T_m_ was due to Fab thermal instability (Fig. 3a; Supplementary Figure 1).

We conclude that the decrease in I4 thermostability was a result of destabilizing mutations acquired early in the affinity maturation process (I8 to I4) and was associated with the acquisition of ^14^S….GG^31^ sequences in the V_H_ FWR1. Although the above FWR1 motif accounted for much of the thermal destabilization effect, other residues outside this region may be involved. However, unlike the ^14^S…GG^31^ residues (Figs. 2, 3a), V_H_ mutations of other residues outside the binding site were not fixed in the lineage and variation in the mutational changes was observed as maturation progressed^24^. Thus, the thermally destabilizing P14S/S30G/S31G substitutions were necessary mutational changes for development of CH103 bnAbs.

### Thermostability and gain-of-function in the CH103 lineage

**Fig. 4:**
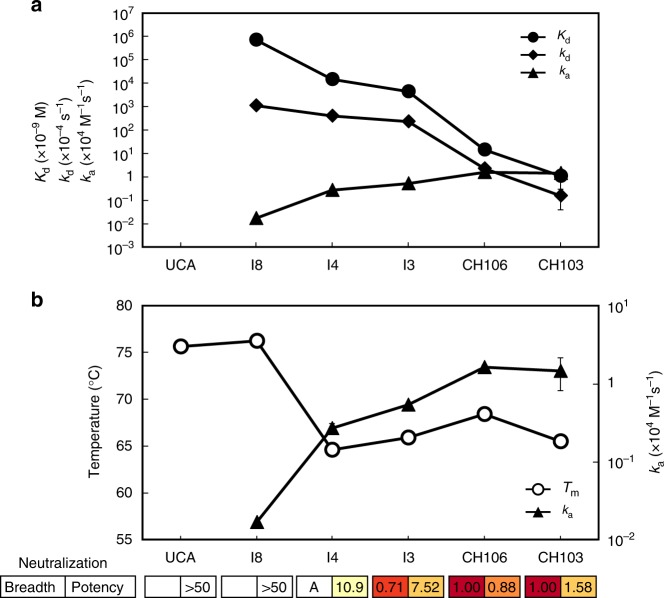
Affinity and thermal stability trade-off in the CH103 bnAb lineage. **a** Apparent affinity (*K*_d_, circles) and kinetic association (*k*_a_, triangles) and dissociation rates (*k*_d_, diamonds) of CH103 lineage mAbs binding to HIV-1 Env B.63521 gp120 (heterologous Env) show changes in kinetic rates during affinity maturation. CH106 and CH103 mAbs bound to Env with high affinity (*K*_d_ < 20 nM) and demonstrated breadth in neutralization^24^. **b** The initial improvement in the association rate (*k*_a_, y_2_-axis, closed triangles) for binding to heterologous Env (B.63521) occurs at the I8–I4 transition and coincides with the observed reduction in antibody thermal stability (T_m_, y_1_-axis, open circles). Data are plotted as the mean and standard deviation from a minimum of two replicate measurements. Certain error bars are smaller than the data markers at this scale. Boxes depict the neutralization breadth and the geometric mean potency (IC_50_, μg/mL) calculated from previously published data^24^. A value of 50 was used for those with IC_50_ > 50. An “A” for the neutralization breadth indicates the antibody neutralizes autologous virus only

A critical feature in a bnAb lineage is the ability to bind to diverse clades of viral antigens, and therefore, a key step in bnAb development is a gain-of-function with respect to binding Env variants. The CH103 UCA binds to the transmitted/founder (TF) CH505 Env but not to heterologous Env^24,26^. To address how gain in affinity for heterologous Env (B.63521) developed in the lineage, we analyzed the kinetic rates (association rate, *k*_a_ and dissociation rate, *k*_d_) of each antibody in the CH103 lineage. We observed that CH103 lineage binding affinity enhancement (*K*_d_ > 100 µM to 1 nM) involved distinct kinetic rates improvement at different stages of affinity maturation (Fig. 4a; Supplementary Table 5). In the early stage (I8 to I3), affinity enhancement was primarily due to improved *k*_a_ (>50-fold faster), while the late stage maturation (I3 to mature mAbs) was dominated by improved *k*_d_ (100-fold slower). The earliest gain in affinity to heterologous Env was observed in I4 primarily due to improved *k*_a_. The ~16-fold increase in *k*_a_ of I4 (2.7 × 10^3^ M^−1^ s^−1^) when compared to that of the germline-proximal intermediate I8 (0.17 × 10^3^ M^−1^ s^−1^) contributed to an order of magnitude difference in *K*_d_ between the two intermediates. The observed difference in the kinetic rates of CH103 lineage antibodies binding to heterologous Env is in contrast to the kinetic rates of binding to the autologous TF Env^26^. The faster *k*_a_ (>10^4^ M^−1^ s^−1^) observed for UCA binding to the TF Env was maintained in the lineage and the order of magnitude gain in affinity (*K*_d_ = 430 nM (UCA) to 40 nM (CH103)) (Table 2; Supplementary Figure 2, Table 5) was due to reduction in dissociation rates (*k*_d_ = 101 × 10^−4^ s^−1^ (UCA) to 2.6 × 10^−4^ s^−1^ (CH103)). These results demonstrated that the *k*_a_ increase observed early in the lineage is associated with gain-of-function with respect to binding heterologous Env (Fig. 4a). Since I4, with a substantial increase in mutation frequency (Fig. 2a), showed lower T_m_ than I8 and UCA, there is an inverse relationship between gain-of-function (heterologous Env binding) and antibody thermostability (Fig. 4b). However, in the later intermediates and mature antibodies, when the affinity for the heterologous Env increases further, the T_m_ of the antibodies are held above the threshold T_m_ of the earlier intermediate I4 (Fig. 4). Thus, the I8–I4 transition is the key initiation step in which selection of the intermediate subsequently allowed later mutations to enhance affinity to heterologous Env and develop neutralization breadth without further detriment to antibody thermostability.

In the lower branch of the CH103 lineage tree, the germline FWR1 P14 residue was retained and the HCDR1 proximal S30 residue was mutated to T in each of the matured non-bnAbs (V_H_ mutations 7.7-15.2%) (Fig. 2a, c). Binding to the heterologous Env (B.63521) was orders of magnitude weaker (*K*_d_: 1–15 µM) for the mAbs in the lower branch when compared to those in the CH103 bnAb branch (*K*_d_: 1–14 nM). Furthermore, the matured lower branch antibodies did not develop neutralization breadth^24^. Although a drop in T_m_ was observed in the most mutated antibody when compared to either intermediate I7 or UCA, there was a concomitant decrease in T_onset_ (Supplementary Figure 1), indicating that there was overall structural instability in the mature antibody that was distinct from the P14S/S30G/S31G associated Fab destabilization observed in the CH103 branch of the lineage tree. These results show the importance of the early selection of the mutations in I4 and the subsequent evolvability of higher affinity antibodies and the acquisition of neutralization breadth.

### The P14S/S30G mutations alter Fab orientation preferences

**Fig. 5:**
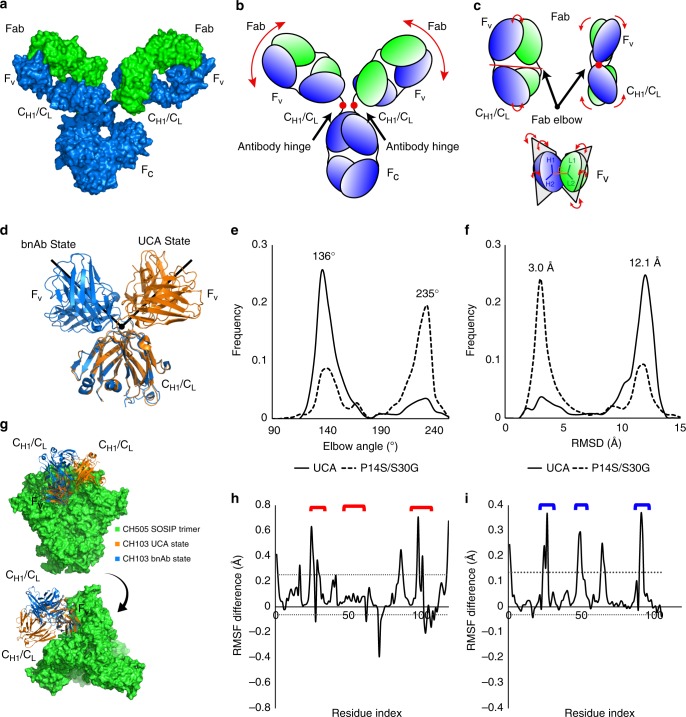
Antibody structure and CH103 Fab elbow flexibility. **a** Surface representation of an IgG antibody. The heavy chain (blue) and light chain (green) pair in the Fab region made up of the antigen binding F_v_ region and the C_H1_/C_L_ region leading to the heavy chain pairing F_c_ region. **b** Cartoon representation of an IgG antibody. The portion of the structure connecting the C_H1_/C_L_ region to the F_c_ region is a site of flexibility termed the antibody hinge. **c** Top: Additional antibody hinge point between the Fab F_v_ and C_H1_/C_L_ region termed the Fab elbow. Bottom: Fab F_v_ V_H_ and V_L_ orientation flexibility about two planes. **d** Structural alignment of the CH103 and CH103 UCA crystal structures’ (PDB ID 4JAM chains H and L and 4QHK chains O and P, respectively) C_H1_/C_L_ region depicting the elbow angle differences. **e** Elbow angle distribution for the five 1 μs simulations of the CH103 UCA (solid) and the CH103 UCA-P14S/S30G mutant (dashed). **f** RMSD to the CH103 bnAb (PDB ID 4JAM) distribution for the five 1 μs simulations of the CH103 UCA (solid) and the CH103 UCA-P14S/S30G mutant (dashed). **g** Structural superposition of the CH103 UCA and CH103 UCA-P14S/S30G onto a CH505TF SOSIP timer homology model based upon the CH103 bound gp120 configuration. **h** Aggregate simulation V_H_ RMSF difference between the CH103 UCA-P14S/S30G mutant and the CH103 UCA. Dotted lines indicate one standard deviation from the mean. Red brackets indicate HCDR positions. (**i**) Aggregate simulation V_L_ RMSF difference between the CH103 UCA-P14S/S30G mutant and the CH103 UCA. Dotted lines indicate one standard deviation from the mean. Blue brackets indicate LCDR positions

While paratope mutations are known to impact Ab loop flexibility, additional sites affecting structural flexibility include the well-known antibody hinge point (Fab-F_c_) as well as the Fab elbow joint (F_v_-C_H1_/C_L_) (Fig. 5a–c). A structural alignment of the C_H1_/C_L_ regions of the available CH103 bnAb and UCA crystal structures indicated a marked shift in the orientation of the F_v_, relative to the C_H1_/C_L_, via rigid body rotation of the two regions about the elbow hinge point yielding elbow angles of 132° and 245° for the UCA and CH103, respectively (Fig. 5d). Since the CH103 P14 residue is in the Fab elbow region, we hypothesized that the acquisition of the P14S mutation, together with the FWR1 S30G mutation, increased flexibility at the I4 Fab elbow and gave rise to increased CDR and V_H_-V_L_ orientational flexibility. We employed long timescale (µs) molecular simulations to elucidate the source of the apparent increase in CH103 lineage antibody flexibility observed in the thermal denaturation experiments. Results from five 1 µs simulations of both the UCA and UCA-P14S/S30G Fabs indicated marked shifts between the UCA and bnAb elbow orientation states (Fig. 5e, f; Supplementary Figure 3, Movie 1). The distribution in elbow angle states for the UCA indicated it strongly favors its initial UCA state, accounting for 82.6% of the simulation time, while the UCA-P14S/S30G mutant displayed a bimodal distribution in elbow angle states, accessing the CH103 bnAb state for 64.7% of its simulation time (Fig. 5e–f; Supplementary Figure 4). Additional changes in the V_H_-V_L_ orientation toward that of the bnAb orientation accompanied these mutations (Supplementary Figure 5). Remarkably, these mutation-induced changes in orientation resulted in major increases in flexibility of CDR loops in both chains and limited potential C_H1_/C_L_ clash with the adjacent protomer in the CH505 Env trimer (Fig. 5g–i). In order to verify the apparent shift in elbow angle preference in these simulations, we used structures of the shifted elbow angle state from the previous simulations of both the CH103 UCA and CH103-P14S/S30G. We extracted Fab coordinates from each individual simulation in which the elbow angle shifted to beyond 180 degrees (Supplementary Figure 6). The differences observed in the simulations of these constructs is consistent with the measured changes in T_m_ and with the V_H_-V_L_ orientation changes in the bnAb crystal structure^19^. The details of these domain changes and their residue influences are discussed further in the supplemental material (Supplementary Discussion, Fig. 3–7). Together, these results suggest that the dramatic shift in the domain interaction network of the CH103 UCA with the destabilizing P14S and S30G mutations was likely critical for shaping downstream affinity maturation of the CH103 bnAb lineage.

### Thermostability and elbow angles in a glycan-V3 bnAb lineage

In the glycan-V3 bnAb lineage DH270^25^, the UCA and early intermediate (I) antibodies exhibited higher T_m_ than the mature bnAbs (Fig. 3b). As observed in the CH103 lineage, the germline-proximal intermediate (I4) of the DH270 bnAb lineage had similar T_m_ to that of the DH270 UCA, and the earliest, and largest, decrease in T_m_ was observed in the next intermediate I2, followed by an additional decrease in I1 and a relatively small increase in the mature DH270.6 bnAb (Fig. 3b; Supplementary Table 6). Similarly, in the lower branch of the DH270 lineage that diverged at I4, the intermediate I3 showed a marked reduction in T_m_ that was further reduced in the DH270.2 bnAb (T_m_ = 59.3 °C) (Supplementary Table 6). Thus, in a second bnAb lineage of differing specificity (glycan-V3 DH270 versus CD4-bs CH103) we observed that thermal destabilization first occurred in an earlier intermediate and thereafter the lower T_m_ was maintained through to the affinity-matured bnAbs (Fig. 3).

We next asked whether the dramatic reduction in T_m_ (12.7 °C) between the DH270 UCA and I2 (Fig. 3b) could have also involved shifts in the elbow angle distribution. As in the CH103 lineage, an elbow region mutation in V_H_ FWR1 (V11M) accompanied this initial drop in T_m_. The aggregate I2 MD simulation elbow angle distribution was markedly shifted in the presence of the I2 mutations with a concomitant increase in HCDR3 flexibility (Supplementary Figure 8, Movie 2). Similar to the CH103 lineage simulations, the altered elbow angle distribution was associated with changes in the V_H_-V_L_ orientations as well as markedly increased flexibility in HCDR3 (Supplementary Figures 8–9). The DH270 UCA displayed a propensity to sample wide and narrow elbow angles with a relatively flat distribution at wider angles, indicating greater flexibility at those angles. Conversely, the DH270 I2 elbow angle was less dynamic and displayed a strongly bimodal distribution with relatively small shoulders. The change in elbow angle distribution was further validated using five 1 µs simulations of coordinates extracted from each simulation in which the elbow angle transitioned. In agreement with the initial simulations, the DH270 UCA displayed a more diverse set of elbow angle states, and the I2 simulations again displayed a bimodal distribution. (Supplementary Figure 10). Similar to the CH103 lineage simulations, the DH270 lineage simulations further demonstrated that changes in elbow angle distributions are associated with changes in paratope flexibility.

### Elbow mutations are common among HIV-1 bnAbs

We next sought to determine whether mutations in the elbow region, including the V_H_ ball-and-socket residues 11, 110, and 112, are common among the various classes of bnAbs (Table 3). The evaluated residues were selected based on the correlation between the CH103 UCA-P14S/S30G elbow joint destabilization and the CH103 T_m_ data and included ball-and-socket adjacent and neighboring residues between V_H_ positions 8–14 and 107–113 (Supplementary Figure 11). Sequence analysis revealed elbow mutations are common, with ~91% of the bnAbs containing mutations in the elbow region and ~55% of the bnAbs containing mutations in the ball-and-socket residues (Table 3). The CD4-bs bnAbs displayed the greatest degree of elbow region mutation, consistent with the potential role of such mutations in the development of neutralization breadth, as demonstrated in the CH103 lineage. While elbow mutations tend to increase with increasing mutation rates in the FWR, several bnAbs (e.g., BF520) with relatively limited V_H_ FWR mutation rates (BF520 = 5.7%) had numerous elbow mutations (Table 3), and in two bnAb lineages (i.e., CH103 and DH270 lineages), the destabilizing elbow region mutations were selected in germline-proximal intermediates with relatively lower numbers of V_H_ mutations. Analysis of V_L_ elbow region sequences revealed fewer mutations, suggesting mutation in the V_H_ elbow region is the dominant source of elbow conformational control in bnAb development (Supplementary Table 7).

**Table 3:** BnAb elbow region mutation frequencies

bnAb	Epitope	% FWR	% Elbow	V_H_ Elbow Mutations	V_H_***	V_H_*	V_H_
8ANC131	CD4 BS	31	42.9	A9G,E10G*,V11L***,T107S,T110I***,S112T***	3	1	6
CH103	CD4 BS	20.7	42.9	L11V***,P14S,T107S,T110S***,S112T***,S113A*	3	1	6
VRC13.01	CD4 BS	44.8	57.1	A9T,E10A*,V11M***,K13S,P14L,L108F,T110R***,S113P*	2	2	8
N6	CD4 BS	31	42.9	A9T,E10A*,V11M***,M108T,T110V***,S113A*	2	2	6
VRC01	CD4 BS	34.5	35.7	A9G,E10Q*,V11M***,L108P,T110I***	2	1	5
VRC16.01	CD4 BS	19.5	28.6	L11F***,Q13K,T110I***,S113A*	2	1	4
CH235.12	CD4 BS	36.8	50	A9G,E10G*,K13R,P14L,T107S,L108P,T110I***	1	1	7
VRC27.01	CD4 BS	34.5	35.7	A9P,E10Q*,K12R*,L108R,T110V***	1	2	5
VRC-CH31	CD4 BS	29.9	28.6	E10A*,K12R*,L108P,T110V***	1	2	4
VRC18.02	CD4 BS	29.9	28.6	A9N,E10Q*,K12R*,T110I***	1	2	4
NIH45-46	CD4 BS	35.6	35.7	A9G,E10Q*,V11M***,T107A,L108P	1	1	5
1B2530	CD4 BS	32.2	28.6	A9T,E10A*,K12R*,T107S	0	2	4
3BNC117	CD4 BS	28.7	21.4	E10A*,K12T*,L108Q	0	2	3
3BNC60	CD4 BS	32.2	21.4	E10A*,K12T*,L108Q	0	2	3
8ANC195	gp120-gp41	35.6	28.6	A9T,V109I*,T110S***,S113A*	1	2	4
35O22	gp120-gp41	26.4	14.3	V11L***,V109L*	1	1	2
PGT151	gp120-gp41	16.1	0	–	0	0	0
VRC34	gp41	19.5	7.1	T108S	0	0	1
DH511.2	MPER	19.5	7.1	T110I***	1	0	1
2F5	MPER	11.5	21.4	T10P*,T107I,V111I*	0	2	3
4E10	MPER	10.3	7.1	K13R	0	0	1
10E8	MPER	20.7	0	–	0	0	0
PGDM1400	Glycan V1/V2	32.2	21.4	A9P,K12R*,T108A	0	1	3
PGT145	Glycan V1/V2	20.7	7.1	T108A	0	0	1
PG9	Glycan V1/V2	13.8	0	–	0	0	0
CH01	Glycan V1/V2	26.4	21.4	G9A,G10N*,T110S***	1	1	3
BF520	Glycan V3	5.7	21.4	V11M***,K13M,T110S***	2	0	3
DH270.6	Glycan V3	17.2	28.6	E10Q*,V11M***,K13N,V109L*	1	2	4
PGT128	Glycan V3	24.1	21.4	G10T*,K13E,P14A	0	1	3
PGT135	Glycan V3	21.8	14.3	T107V,L108Q	0	0	2
PGT121	Glycan V3	20.7	7.1	T108Q	0	0	1
2G12	Glycan V3	29.9	21.4	P14A,M108V,S113*	0	1	3

V_H_ framework and elbow region mutation frequencies (%) are relative to the unmutated common ancestor. Asterisks indicate whether residues occur in the ball-and-socket (***) or are ball-and-socket adjacent (*). The V_H_ list includes all residues in the elbow region.

### Elbow mutations are uncommon in weakly neutralizing Abs

We next asked whether weakly neutralizing HIV-1 antibodies also frequently accumulate V_H_ elbow mutations. Comparison of nine previously investigated mature non-bnAbs to their germline encoded sequences revealed that, unlike bnAbs, V_H_ elbow mutations are relatively uncommon (Supplementary Table 8). As mutation frequency in these antibodies is typically lower than their bnAb counterparts, we compared the V_H_ framework and elbow mutation frequencies of these non-bnAbs to the frequencies in the CH103, DH270, CH235, and N6 bnAb lineages. In spite of similar framework mutation frequencies between several bnAb lineage members and non-bnAbs, elbow mutation frequencies were generally higher in bnAbs than non-bnAbs at similar framework mutation frequencies (Supplementary Figure 12). That is, the bnAbs display a tendency of disproportionately accumulating elbow mutations at overall comparable framework mutation frequencies.

In comparison, the gp120 V2 non-bnAb CH58, isolated from an RV144 ALVAC/AIDSVAX vaccinee, that binds to the same region as glycan-V2 bnAbs^27^, had no elbow region mutations, and therefore, we hypothesized that major shifts in the elbow angle distribution would not occur. MD simulations showed that unlike the UCAs of CH103 and DH270  bnAbs, the CH58 UCA remains largely trapped in its initial elbow angle state (Supplementary Figure 13a, Movie 3). The mature CH58 displays a wider elbow angle distribution than its UCA but also fails to access wide elbow angle states (Supplementary Figure 13). Nevertheless, the T_m_ for CH58 (63.0 °C) was lower than its respective UCA (69.4 °C), and MD simulations revealed shifts in the V_H_-V_L_ orientations with an increase in HCDR3 flexibility (Supplementary Figures 14–15). While CH58 demonstrates a reduced thermostability relative to its UCA, it lacks FWR elbow mutations and does not exhibit the characteristic elbow angle flexibility observed in the bnAbs. Interestingly, a related V2 non-bnAb CH59, isolated from the same vaccinee as CH58^27^, also showed no elbow region mutations but demonstrated increased T_m_ (73.7 °C) relative to its UCA (67.4 °C) (Supplementary Figure 15). It is likely that the observed T_m_ differences in CH58 and CH59 are a consequence of different antibody structural dynamic characteristics and/or a means of thermal stabilization/destabilization that does not involve elbow flexibility.

### Elbow mutations and neutralization breadth in HIV-1 bnAbs

In order to determine whether elbow mutations accompany increases in heterologous breadth and potency, we examined the timing of the selection of elbow region mutations during bnAb maturation. In the CH103 lineage, the first thermally destabilizing elbow region mutation (P14S) was selected in I4, which neutralizes the Tier 2 autologous virus (Supplementary Figure 16) and binds weakly to heterologous Env protein but does not neutralize heterologous viruses (Fig. 4). Nevertheless, the initial selection of the destabilizing elbow mutation P14S in I4 was required for interdomain flexibility and the subsequent development of neutralization breadth in the lineage. As such, we observed that a progressive increase in neutralization breadth and potency is associated with selection of additional elbow mutations in the later intermediates which are retained in each of the bnAbs, both in the ball-and-socket and in adjacent residues (Supplementary Figure 16). In contrast, antibodies in the lower branch of the CH103 lineage with no destabilizing FWR elbow region mutations do not neutralize heterologous virus, and even with ~15% V_H_ mutations, the most somatically mutated mAb isolated in this branch only weakly neutralizes the autologous virus (Supplementary Figure 16).

The association of FWR elbow region mutations and neutralization breadth was also observed in the glycan-V3 bnAb DH270. Specifically, the first elbow mutation (V11M) is in I2, the intermediate at which a reduction in T_m_ was first observed, which coincides with the acquisition of neutralization breadth in the lineage (Supplementary Figure 17). Further increases in breadth and potency among DH270 bnAbs were associated with the acquisition of additional mutations in residues adjacent to the ball-and-socket region (K13N, V109L). Considering that our simulation and experimental results indicate the potential for elbow mutations to have profound impacts on antibody physical properties, elbow mutations likely had an impact on the development of heterologous breadth in both the CH103 and DH270 bnAb lineages. Consistent with these observations, elbow mutations in the CD4-bs N6 bnAb lineage between intermediates I2 and I3 were associated with dramatic increases in neutralization breadth and potency^28^.

Interestingly, in another CD4-bs bnAb lineage (CH235)^24,26^, the CH235 bnAb, which accumulated less mutations and displayed narrower neutralization than more mutated clone members^26^, contained a single elbow mutation at position 113 (Supplementary Figure 18), adjacent to the ball-and-socket region^26^. This particular position, 113, is distal to the elbow region and solvent exposed, limiting its ability to influence the overall Fab architecture. However, the more affinity matured members of the lineage, CH235.9 and CH235.12, which reached broader neutralization, contained five and seven mutations in the elbow region, respectively, which included both a P14L and a ball-and-socket residue mutation (T110I) (Table 3). Therefore, we predicted that, while CH235 was unlikely to be thermally destabilized relative to the UCA, CH235.9 and CH235.12 would likely display a markedly reduced T_m_. Indeed, the T_m_ for CH235 (76.7 °C) was higher than that of the UCA (74.0 °C), and as predicted, the T_m_ values for CH235.9 and CH235.12 were greatly reduced to 66.9 and 67.7 °C, respectively (Supplementary Figure 19). The reduced T_m_ in the later antibodies is in accord with our observations in the CH103 and DH270 lineages and is similarly associated with an increase in neutralization breadth from 18% in CH235 to 77% in CH235.9 and 90% in CH235.12^26^.

### Effect of reversion mutations on neutralization

To directly determine the functional impact of destabilizing as well as Fab elbow region mutations on neutralization potency and breadth, we produced revertant mutants of key early intermediates and affinity-matured bnAbs of both the CD4-bs CH103 and the glycan-V3 DH270 bnAb lineages. We measured the effect of reversion mutations on thermostability and neutralization potency for each of the intermediates and bnAbs.

**Fig. 6:**
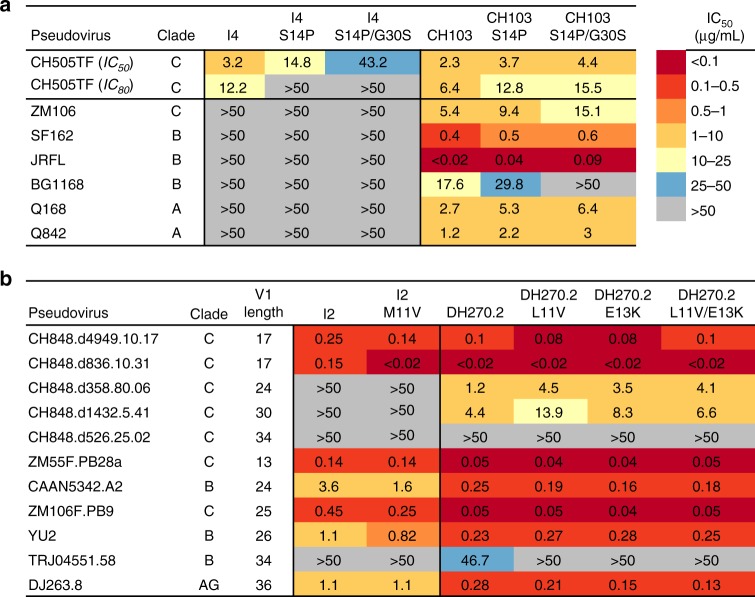
Effect of reversion mutations on neutralization potency and breadth. **a** Antibody neutralization of the wild-type and reversion mutants of the CH103 lineage intermediate I4 and the CH103 bnAb against autologous and heterologous Env-pseudoviruses. **b** Antibody neutralization of the wild-type and reversion mutants of the DH270 lineage intermediate I2 and the DH270.2 bnAb against autologous and heterologous Env-pseudoviruses. Antibody neutralization was measured by a TZM-bl cell-based assay using a select panel of geographically and genetically diverse Env-pseudoviruses. All values are IC_50_ (µg/mL) unless otherwise noted

For the CH103 lineage, we produced two CH103 germline-revertant mutants, a single-residue mutant with the S14P mutation (CH103-S14P) and a double mutant (CH103-S14P/G30S) with both S14P and G30S mutations. Similarly, for the intermediate I4, a single mutant I4-S14P and a double mutant I4-S14P/G30S were produced. CH103 I4 revertant mutants showed improved thermostability compared to WT (ΔT_m,I4-S14P_ = + 2.4 °C; ΔT_m,I4-S14P/G30S_ = + 3.8 °C) and an overall 2.5-fold reduction in affinity for the autologous CH505TF SOSIP trimer (*K*_d,I4_ = 60 nM, *K*_d,I4-S14P/G30S_ = 160 nM) (Table 1; Supplementary Tables 2-3). CH103 revertant mutants had marginally improved thermostability compared to WT (ΔT_m,CH103-S14P_ = + 0.9 °C; ΔT_m,CH103-S14P/G30S_ = + 1.1 °C) but a 10-fold reduction in affinity for the autologous CH505TF SOSIP trimer (*K*_d,CH103_ = 16 nM, *K*_d,CH103-S14P/G30S_ = 150 nM) (Table 1; Supplementary Tables 2-3). Reversion of the two destabilizing residues that increased elbow flexibility in the CH103 lineage had nominal effect on the autologous CH505TF neutralization potency of the CH103 bnAb (Fig. 6a, IC_80_ = 6.4, 12.8, and 15.5 µg/mL for CH103, CH103-S14P, and CH103-S14P/G30S respectively). However, the neutralization potency of the single S14P mutant of CH103 was reduced against two of the Tier 2 heterologous pseudoviruses (ZM106 and BG1168), and the double mutant CH103-S14P/G30S failed to neutralize the clade B BG1168 virus (Fig. 6a), indicating that the destabilizing mutations were important for neutralization of heterologous viruses. Reversion of mutations in the intermediate I4, which only neutralized the autologous CH505TF virus (IC_50_ = 3.2 µg/mL), markedly weakened the neutralization potency. The single-residue reversion mutant I4-S14P neutralized CH505TF virus with about 5-fold weaker potency (IC_50_ = 14.8 µg/mL, IC_80_ > 50 µg/mL), and almost complete loss of neutralization was observed with the double mutant I4-S14P/G30S (IC_50_ = 43.2 µg/mL, IC_80_ > 50 µg/mL, Fig. 6a). Thus, reversion of the two destabilizing residues that allowed greater Fab elbow flexibility in the CH103 bnAb lineage nearly abolished the ability of I4 to neutralize.

We previously reported that the development of the V3 and N^332^-glycan-dependent DH270 bnAb lineage involves overcoming evolutionary hurdles imposed by the steric constraints of V1 length and the adaptation to V3 glycans^25^. Thus, a hallmark of DH270 bnAb development is that the neutralization breadth in the DH270 lineage was associated with the ability to neutralize viruses of varying gp120 V1 lengths^25^. The I2 intermediate in the DH270 lineage potently neutralized multiple autologous virus quasi-species with short V1 (17 aa) and the single residue reversion mutation M11V did not impact the ability of I2 to neutralize (Fig. 6b). Neither the WT nor the revertant mutants of I2 neutralized CH848 viruses with longer V1 (Fig. 6b). However, the introduction of elbow mutations in a more affinity-matured member of the lineage (DH270.2) resulted in a 3–4 fold reduction in neutralization potency against Tier 2 pseudoviruses with longer V1 (>17 aa) that were neutralized with moderate potency (IC_50_ > 1  µg/mL) by WT DH270.2. The predominant effect on DH270.2 neutralization potency was observed with the single residue L11V reversion mutation (Fig. 6b). That reversion mutations in the broadest and most affinity matured member of the DH270 lineage (DH270.6) had modest impact on T_m_ and no impact on its neutralization potency against either autologous or heterologous viruses (Supplementary Tables 9–10) suggests that the additional affinity maturation of DH270.6 conferred the ability to more efficiently adapt to variation in V1 length. Thus, in the glycan-V3 DH270 lineage, the functional impact of the reversion of elbow region mutations was only observed for DH270.2 bnAb against the more difficult-to-neutralize viruses with longer V1 length.

Overall these results show that the selection of destabilizing mutations contribute to Fab elbow flexibility and are important for autologous neutralization early in the CD4-bs CH103 lineage and for heterologous neutralization for the affinity-matured bnAb. For the glycan-V3 DH270 lineage, the impact of the reversion mutations was observed only in one branch of the lineage and against viruses that were relatively more difficult to neutralize.

## Discussion

We have studied the thermostability of bnAbs with different specificities for HIV-1 Env gp120 and gp41 epitopes and report that all major classes of bnAb development are associated with thermal destabilization. Using CD4-bs and glycan-V3 bnAb lineages with inferred UCAs and intermediates, we show that thermostability is selected early in affinity maturation, at an intermediate state when both affinity gain for diversity in antigen recognition and thermal destabilization are concurrently achieved. Once selected, the destabilizing mutations are fixed in the lineage and the lower thermostability is maintained in the affinity-matured bnAbs, indicating that selection of key destabilizing mutations are important for bnAb development. In the CD4-bs CH103 bnAb lineage, we identified FWR mutations that contributed to thermal destabilization, and we show that a key mutated residue resides in the Fab elbow region and facilitates the development of paratope plasticity for heterologous Env recognition and development of neutralization breadth. Using long timescale (µs) molecular dynamics simulations, we demonstrated that the V_H_ FWR elbow mutations impact inter-domain dynamics due to altered Fab elbow and V_H_-V_L_ orientational flexibility and similar mutational changes are required in several key bnAb lineages. We report that, unlike weakly or strain-specific neutralizing antibodies, elbow region mutations are frequently observed in HIV-1 bnAbs, and are selected consistently in bnAbs that target the CD4-bs and glycan-V3. In four bnAb lineages, we charted the progressive selection of Fab elbow region mutations and showed that selection of elbow mutations coincided with increases in heterologous neutralization breadth and potency. In contrast, antibodies that matured in the CH103 bnAb lineage without key elbow mutations failed to neutralize heterologous viruses even with high V_H_ somatic mutation frequencies. Reversion of destabilizing mutations to the respective germline-encoded amino acids negatively affected the neutralization potency of the CH103 bnAb against heterologous viruses and almost abrogated the ability of the early intermediate to neutralize the autologous virus. In the glycan-V3 DH270 lineage, the impact of the reversion mutations was limited to neutralization of viruses with longer V1 lengths by the DH270.2 bnAb in one branch of the lineage. Together, these results show the molecular basis for the selection of destabilizing mutations in the Fab elbow region during affinity maturation of two classes of HIV-1 bnAb lineages (CD4-bs, glycan-V3) and indicate that selection of such mutations should be considered in HIV-1 immunogen design.

Our molecular simulation results suggest a major contributor to the early loss in thermostability in the CH103 lineage is related to changes in the Fab’s ability to sample differing elbow and V_H_-V_L_ orientation states. Previous MD investigations have demonstrated similarly dynamic elbow flexibility and that both elbow angle shifts and changes in V_H_-V_L_ orientations are attributable to framework residue mutations^14,29^. This suggests that such flexibility is not unusual and likely plays an important role in enhancing antibody affinity and stability. However, the implications of any particular elbow mutation in the stabilization or destabilization of the V_H_ elbow is likely context dependent, and their specific roles in bnAb development are not well defined. In this study, our MD simulations show that, certain V_H_ sites, particularly in the ball-and-socket and FWR1 P14, are expected to be more sensitive to mutation-induced alteration of elbow flexibility. Considering the early selection and extent of elbow mutations in CD4-binding site and glycan-V3 bnAbs, changes in elbow dynamics likely played an important role in shaping downstream development of heterologous breadth. Indeed, comparison of simulation results for the DH270 UCA and I2 here indicate a potential role for the V11M mutation in altering the elbow angle conformation. Alternatively, the non-bnAb CH58, with no elbow mutations, displayed no change in the propensity to sample wide elbow angles. Together, these results further demonstrate the ability of paratope distant residues to have a major impact on antigen recognition and affinity maturation and suggest several bnAb classes have taken advantage of such properties in the development of heterologous Env recognition.

Mutations in the Fab elbow region are commonly observed in HIV-1 bnAbs and the frequency of elbow region mutations were higher for certain classes of bnAbs, particularly those that targeted the CD4-bs or the glycan-V3. A glycan-V3 bnAb, 2G12, acquired mutations in the V_H_/V_H_’ interface and elbow regions and configured a multivalent Fab domain-swapped binding site^30^, indicating that elbow region mutations work in concert with other key mutations. Antibody paratope configurations resulting in both rigidification and increased flexibility have also been observed^31^. However, the path to such binding site configuration could require early selection of key mutations that are required for optimization of both structure and function. Our studies with revertant mutants of an early intermediate and the affinity-matured bnAb of the CD4-bs CH103 lineage showed that the impact on neutralization potency and breadth was dependent on affinity maturation stage. While the selection of the destabilizing mutations in the CH103 lineage coincided with increased heterologous Env affinity, the marked loss of autologous neutralization in the revertant mutants of the intermediate I4 indicated that these mutations were important for autologous neutralization prior to development of neutralization breadth. The impact of the reversion mutations in the affinity-matured CH103 bnAb was observed only against heterologous and not autologous viruses. Thus, the destabilizing elbow region mutations are important both at an early maturation stage when the intermediate has not yet achieved sufficient affinity for potent neutralization and later in the affinity-matured bnAb for difficult-to-neutralize heterologous viruses. In the DH270 lineage, the impact of reversion mutations was limited to DH270.2 that branches out from I3, an earlier intermediate that unlike I2 has limited breadth and potency and no elbow region mutations. The intermediate I2, unlike the I4 in the CH103 lineage, shows breadth in neutralization and potently neutralizes autologous viruses with relatively shorter V1 lengths. The DH270 I2, therefore, represents an intermediate at a more advanced maturation stage, and as observed with the most potent DH270.6 bnAb, reversion of elbow region mutations had no impact on neutralization potency against the tested panel of viruses. Thus, the impact of reversion mutations on neutralization was limited to the bnAb in the lower branch of the DH270 lineage and against viruses that were relatively more difficult to neutralize. The observed differences in the two evolutionary branches of the DH270 lineage is not unique and has been described for the glycan-V3 PGT121 lineage that showed a selective preference for glycans on different residues for antibodies on the two branches^32,33^. Our current studies addressed only the impact of V1 length on neutralization potency of selected DH270 mutants and therefore, further studies with glycan mutants might provide a more complete understanding of the role of the elbow region mutations for antibodies in the upper branch of the DH270 lineage.

The decrease in thermostability and altered elbow region flexibility could be selected against under normal antibody development, therefore suggesting a barrier for bnAb elicitation that needs to be accounted for in vaccine design strategies. A common trait associated with all major classes of matured HIV bnAbs is polyreactivity and/or autoreactivity^34,35^. While CH103 bnAbs were polyreactive, the UCA and the intermediates with destabilizing elbow mutations were not self or polyreactive^24^, suggesting that selection of interdomain flexibility itself would not be disfavored. However, the impact of antibody elbow flexibility on B cell signaling and survival is unclear. While murine V_H_ chain usage with a more flexible elbow is positively selected in mature B cells^36^, elbow conformational flexibility can impact initiation of cell signaling if antigen binding induces conformational changes and BCR reorganization is required for full activation^37,38^. It is likely that vaccine design strategies aimed at recapitulating the first step of increased global Fab flexibility could increase the probability of selection of the more favored bnAb precursor pools and allow subsequent selection of key affinity-enhancing mutations that lead to the acquisition of neutralization breadth. As the MD simulations and previous work suggest elbow region mutations alter V_H_-V_L_ orientations and therefore paratope disposition, immunogen design efforts that include steric challenges to the V_H_-V_L_ orientation are likely to select for elbow region mutations via reduced stress at the paratope-epitope interface.

## Methods

### Proteins

Procedures for purification of each of the studied mAbs are described in the previous publications^24,27,39^. Computational inferences and production of the UCA and intermediate antibodies were performed by methods previously described^24,40^. For the CH103 lineage, the individual heavy chains of UCA, I8, I4, I3, and I7 mAbs were expressed with the UCA-light chain. The heavy chains of later intermediates (I2, I1) and lower branch Abs (1A102RI6, 1AZCETI5) were expressed with the I2-light chain. The mature CH103 bnAbs were coupled with somatically-mutated light chains unique to that antibody^24^. All antibody samples were buffer exchanged to the final PBS buffer 137 mM NaCl, 2.7 mM KCl, 10 mM Na_2_HPO_4_, 1.8 mM KH_2_PO_4_, pH 7.4. The final concentrations (per IgG) of the antibody samples were determined from absorption measurements using molar extinction coefficients (M^-1^ s^-1^ per IgG) at 280 nm^41^. Although no aggregates were observed by size exclusion chromatography analysis, as a standard procedure all antibody and protein samples were spun down to remove any potential small scale aggregates prior to performing any measurements.

HIV-1 Env gp120 and gp140 proteins were produced in Freestyle 293 cells (Invitrogen) as previously described^42^. Briefly, HIV-1 gp120 and SOSIP gp140 were purified with lectin affinity and PGT145 affinity respectively. The envelope was size fractionated by gel filtration chromatography to purify monomeric gp120 and trimeric gp140.

### Circular dichroism (CD)

CD melting measurements were carried out on antibody samples in PBS buffer at 0.5–1.5 μM (per IgG molecule) in a 1 mm path length cuvette using the Aviv (Lakewood, NJ) Model 202 spectropolarimeter. CD signals at 208 nm were monitored every 1 °C as the temperature of the antibody samples increased at a ramp rate of 0.5 °C/min. The samples were allowed to equilibrate for two minutes at each temperature before CD signals were recorded at an averaging time of 2 min. The fraction folded after each incremental increase in temperature was calculated using the formula provided by Greenfield^43^. A Savitzky-Golay 13-point quadratic first derivative function was applied to the fraction folded curves to approximate the melting temperature of each antibody.

### Differential scanning calorimetry (DSC)

Antibody thermal denaturation profiles were obtained in HEPES Buffered Saline (HBS; 10 mM HEPES, 150 mM NaCl pH 7.4) at concentrations ranging from 0.2–0.4 mg/mL using the NanoDSC platform (TA instruments; New Castle, DE). All antibody samples were extensively dialyzed into HBS, diluted in filtered dialysate, and degassed for 15 min at room temperature prior to analysis. DSC cells were conditioned with filtered, degassed dialysate prior to sample loading. Antibody samples were heated from 10–20 °C to 100 °C at 1 °C/min under 3 atm pressure using the corresponding dialysate as the reference buffer. The observed, irreversible denaturation profiles were buffer subtracted, converted to molar heat capacity, baseline corrected with a 6^th^-order polynomial, and fit with three Gaussian transition models using the NanoAnalyze software (TA Instruments). The primary transition temperature (T_m_) is reported as the temperature at the maximum observed heat capacity. The transition onset temperature (T_onset_) was calculated as the temperature at which the model sum deviates from the baseline by 2% of its maximum amplitude.

### Surface plasmon resonance (SPR)

SPR measurements of Env protein binding to mAbs were performed on a BIAcore T200 instrument (GE Healthcare) as described earlier^24,44^. Approximately 200–500 RU of each antibody was captured on an anti-human IgF_c_ immobilized sensor surface (CM5, GE Healthcare). Monomeric gp120 proteins were diluted from 0.5 to 200 µg/mL (20–489 nM for CH505TF gp120 and 9–3747 nM for B.63521 gp120) and injected for 5 min at 50 µL/min using the high-performance injection setting. The 5 min injection was followed by a 10 min dissociation period and then a 20s injection pulse of glycine pH 2.0 for sensor surface regeneration. Non-specific binding was accounted for by using in-line reference subtraction of signal on a flow cell captured with control mAb (Synagis, anti-RSV). Binding rate constants (*k*_a_, *k*_d_) were measured following global curve fitting to a Langmuir model. Curve fitting analysis was performed with BiaEvaluation software (GE Healthcare) using a 1:1 Langmuir model to derive rate (*k*_a_, *k*_d_) and dissociation (*K*_D_) constants and results are representative of at least two measurements. A goodness of fit was assessed by examination of randomness in residual plots and chi^2^ values <1 was observed for all affinity measurements.

### Biolayer interferometry (BLI)

BLI measurements of Env protein binding to mAbs were performed on a OctetRed96 instrument (ForteBio). Antibodies were loaded onto anti-human IgG F_c_ capture sensor tips (AHC, ForteBio) by submerging tips in 20 µg/mL mAb in PBS for 300 sec and then washed in PBS for 60 sec at 1000 rpm. Association measurements were performed by submersion in 4.75–475 nM CH505TF SOSIP.664.v4.1 for 400 sec at 1000 rpm, and dissociation measurements were collected by submersion in PBS for 600 sec. Sensor tips were regenerated using a 20 sec submersion in glycine pH 2.0. Non-specific binding was accounted for by using in-line reference subtraction of signal on a sensor tip captured with control mAb (anti-Flu Hemagluttinin Ab82). Curve fitting analysis was performed with the Octet Data Analysis 10.0 software (ForteBio) using a 1:1 model to derive rate (*k*_a_, *k*_d_) and dissociation (*K*_D_) constants.

### Molecular simulation

The unbound crystal structure for the CH103 UCA (PDB ID 4QHK chains O and P) was used for the unmutated CH103 UCA and mutated CH103 UCA-P14S/S30G, Fab simulations^45^. The DH270 UCA.3 (PDB ID 5U15 chains H and L) Fab was used for both the UCA and I2 simulations. The CH58 UCA (PDB ID 4RIR) and CH58 (PDB ID 4HQQ) Fabs were used for CH58 simulations. Missing loops were added to each structure using Modeller with the CH103 heavy chain P14S and S30G mutations and DH270 I2 mutations added using the prepared UCA structures in PyMol^46^. The proteins were solvated in a truncated octahedral box of TIP3P water molecules with a minimum of 12 Å between the protein and solvent box edge using the AmberTools17 Leap program with the system charge neutralized via addition of chlorine atoms^47^. All simulations were run using Amber 16 with the Amber ff14SB forcefield^47,48^. The systems were prepared for production runs over several steps. First, an initial minimization of the solvent-ion system was performed with the protein atoms fixed for 10,000 steps using a non-bonded interaction cutoff of 10 Å. This was followed by minimization of the entire system without restraints on the protein atoms for 10,000 steps. The system was then heated from 0 K to 300 K in the NVT ensemble over a period of 20 ps with a 2 fs time-step using the particle mesh Ewald method using periodic boundary conditions and restraints on the protein atoms^49^. This was followed by 100 ps of unrestrained dynamics at 300 K in the NPT ensemble with the temperature controlled using Langevin dynamics with a frequency of 1.0 ps^−1^ and the pressure maintained at 1 atm using isotropic position scaling with a relaxation time of 2 ps^50^. Hydrogen atoms were constrained using the SHAKE algorithm^51^. Production runs were completed in the NPT ensemble using an 8 Å cutoff for a period of 1 µs for both the UCA and UCA-P14S/S30G with all other parameters identical to the previous 100 ps simulation. Four additional simulations using randomized seed values beginning from the heating run were performed for each construct for a total of 1 µs each. Simulations were visualized and analyzed in VMD and PyMol using tools therein^52^. Alignments and buried surface area calculations were performed in PyMol. Calculation of the root mean square fluctuation of residue alpha carbons was performed in VMD. Elbow angles were determined using PyMol with V_H_-V_L_ angles determined using the ABangle software^53^. The CH505TF Env SOSIP trimer was produced using Modeller with the BG505 SOSIP (PDB ID 5CEZ) used as a template with superpositions of the CH103 UCA and CH103 bnAb Fabs (PDB IDs 4QHK and 4JAN, respectively) performed in PyMol^46^.

### Sequence analysis

Sequences for bnAb Fab V_H_ regions were obtained from GenBank with bnAb UCA sequences determined using Clonanalyst^54^. The sequences were numbered using the Kabat scheme using ANARCI^55^. Framework mutations were determined based upon Kabat assignment of residues in the UCA sequence excluding HCDR residues 31–35, 50–65, and 95–102. Elbow region residues for the V_H_ were defined as residues 8–14 and 107–113. Elbow region residues for the V_L_ were defined as residues 8–15, 82–83, and 105–107. Mutations and mutation frequencies for the framework and elbow regions were determined using custom Perl scripts. Sequence analysis was performed for the following V_H_ gene sequences (GenBank ID): 10E8 (JX645769)^56^, 1B2530 (HE584545)^57^, 35O22 (KM001872)^58^, 3BNC60 (HE584535)^57^, 3BNC117 (HE584537)^57^, 8ANC131 (HE584549)^57^, 8ANC195 (HE584553)^57^, BF520 (KX168065)^59^, CH01 (JQ267523)^60^, CH103 (KC575845)^24^, CH235.12 (KU570042)^26^, DH270.6 (KY354948)^25^, DH511.2 (KY272651)^61^, N6 (KX595108; 27851912), PG9 (GU272045)^62^, PGDM1400 (KP006370)^62^, PGT121 (JN201894)^63^, PGT128 (JN201900)^63^, PGT135 (JN201903)^63^, PGT145 (JN201910)^63^, PGT151 (KJ700282)^64^, VRC-CH31 (JN159435)^65^, VRC01 (GU980702)^66^, VRC34 (KU711816)^67^, NIH45-46 (HE584543)^57^, PGT151 (KJ700282)^64^, VRC13.01 (KP860914)^68^, VRC16.01 (KP860917)^68^, VRC18.01 (KP860916)^68^, VRC27 (26004070)^68^, 2F5^69^, 2G12^69^, 4E10^69^, CH58 UCA (KC417401)^27^, CH58 (KC417393)^27^, CH59 UCA (KC417403)^27^, CH59 (KC417395)^27^, CH103 UCA and intermediates^24^, DH270 UCA-Intermediates^25^, CH235^26^, and CH235.9^26^. Sequences for non-bnAbs were collected from the table in Klein et al.^8^.

### Reporting summary

Further information on experimental design is available in the Nature Research Reporting Summary linked to this article.

## Supplementary information

Supplementary Information

Description of Additional Supplementary Files

Supplementary Movie 1

Supplementary Movie 2

Supplementary Movie 3

Reporting Summary

## Data Availability

The datasets generated and/or analyzed during the current study are available from the corresponding author on reasonable request.
