# Supplementary material for: Selection of immunoglobulin elbow region mutations impacts interdomain conformational flexibility in HIV-1 broadly neutralizing antibodies
Source: Nat Commun. 2019 Feb 8;10:654. doi: 10.1038/s41467-019-08415-7 (PMC6368608; doi:10.1038/s41467-019-08415-7)
Supplement: Supplementary file 1 — Supplementary Information [file 41467_2019_8415_MOESM1_ESM.pdf]

Supplementary Information

Selection of immunoglobulin elbow region mutations impacts interdomain conformational flexibility in HIV-1 broadly neutralizing antibodies

Henderson et al.

Supplementary Discussion

Molecular Simulation of the CH103 UCA and CH103 UCA-P14S/S30G Fabs

The results of the 1 µs simulations of the CH103 UCA andUCA-P14S/S30G mutant Fabs indicated important changes in the F_v region of the protein accompany the increased occupancy of the bnAb elbow angle state, defined here as an elbow angle greater than 180 degrees. The shifted elbow angles of the Fabs align well with the CH103 bnAb with a trajectory minimum root mean square deviation (RMSD) of 1.2 Å and 1.3 Å to the UCA and UCA P14S/S30G, respectively. The regions of the P14S and S30G mutations specifically have an increased RMSF in the mutant as compared to the UCA suggesting the differences in the simulations arise from the increased flexibility at these locations (**Figure 5h-i**). These specific increases in flexibility are consistent with the observed drop in Tm which are accompanied by an overall increase in F_v region flexibility overall (0.7 Å and 0.8 Å RMSF averages for the UCA and UCA P14S/S30G, respectively; **Figure 5h-i**). In order to further validate elbow angle distribution results from the CH103 UCA and CH103 UCA-P14S/S30G 5 µs simulations, we ran four additional 0.25 µs simulations of each construct to provide an additional estimate of the propensity of the UCA and UCA-P14S/S30G to sample the CH103 bnAb state. While both sets display the ability to sample both states (**Supplementary Figure 7a-b**), as in the 5 µs simulations, the UCA-P14S/S30G samples the bnAb state to a greater extent as compared to the UCA (**Supplementary Figure 7c**) with the UCA and UCA-P14S/S30G occupying the bnAb elbow angle state for an average of 18% and 36% of the simulation time, respectively. Stabilizing contacts in each elbow angle state are dominated by interactions between elbow joint residues in the variable and constant regions of either the heavy chain or the light chain (**Supplementary Figure 4**). Specifically, the UCA state is predominantly stabilized by interactions at the heavy chain elbow joint while the bnAb state is predominantly stabilized by the light chain elbow joint. In the UCA, $P14_H$ facilitates a loop turn which helps to stabilize

important interacting elbow joint residues. The $P14_HS$ mutation increases flexibility in this region thereby destabilizing the heavy chain elbow joint contacts thus facilitating an increased probability of transitions. As few contacts exist in the light chain to maintain the UCA state, the F_v and C_{H1}/C_L region are freed to diffuse toward the bnAb state upon destabilization of the heavy chain elbow joint contacts. The specific residues involved in stabilizing the CH103 UCA state at the heavy chain elbow joint include a hydrogen bonding network between the backbone of $G8_H$, $P9_H$, and $T107_H$ with the side chain of $K201_H$, a hydrogen bond between $T110_H$ and the backbone of $E148_H$ and a UCA state stabilizing elbow loop hydrogen bond between $S112_H$ and the backbone of $A114_H$. A key buried leucine at position 11_H rests in a pocket stabilized by interactions near the $P14_HS$ loop turn provides a particularly important contact network providing a total of 742.0 $Å^2$ of buried surface area. This is a position in the V_H $^{10}ELVK^{13}$ motif known to influence Fab elbow stability in the ball-and-socket formed between V_H residues 11, 110, 112, 149 (146 in CH103) and 150 (147 in CH103) in the heavy chain elbow joint.[1] Initiation of transitions from the UCA state to the bnAb state involves destabilization of the $L11_H$ residue resting in this elbow bend pocket. Based upon these simulations, the interactions in the UCA state heavy chain needed to ensure burial of this critical stabilizing $L11_H$ are supported by the $P14_H$ loop. Stabilizing interactions at the light chain portion of the UCA state Fab elbow include a hydrogen bond between $E83_L$ and $K166_L$ and a UCA state, elbow loop stabilizing hydrogen bond between $Q108_L$ and $Y140_L$ with additional residues providing a buried surface area of 771.7 $Å^2$. The bnAb state in the UCA-P14S/S30G light chain is stabilized by several key residues including a network of combined hydrogen bond and electrostatic interactions between $S9_L$, $S12_L$, $E198_L$, and $K110_L$ in addition to a light chain elbow loop stabilizing hydrogen bond between $Y140_L$ and the $G107_L$ backbone. These contacts are nearly identical in the CH103 bnAb except for a $G107_LR$ point mutation. This $R107_L$ in the CH103 bnAb forms a hydrogen bond with $Y140_L$, likely further stabilizing the

light chain elbow loop. A single heavy chain bnAb state, elbow loop stabilizing hydrogen bond occurs between $S113_H$ and the $S173_H$ backbone. While the bnAb state contacts of the CH103 UCA-P14S/S30G mutant are nearly identical to the CH103 bnAb crystal structure (**Supplementary Figure 4**), the absence of additional mutations in the CH103 I4 likely ensures this new state remains comparatively unstable. The buried surface area between the UCA-P14S/S30G C_{H1} and V_H in the CH103 bnAb state was 480.3 $Å^2$ while that of the C_L and V_L was 771.3 $Å^2$. Together, the combined van der Waals, hydrogen bond and electrostatic interactions in the light chain dominate stabilization of the bnAb state with a dramatic decrease in the buried surface area at the V_H elbow joint relative to the UCA. While crystal packing may play a role in the capture of a particular elbow angle [2], these simulations are nevertheless suggestive of differing propensities for the Fabs to occupy different states. Regardless of the degree to which crystallization has influenced the elbow angle of the CH103 UCA, our simulations here indicate that the mutations introduced into the UCA Fab framework region have altered the propensity for transitions between elbow angle states.

While motion of the Fab elbow can be capture well by a single angle term, a complete description of structural differences in the relative orientations of the V_H and V_L require a total of six terms (**Supplementary Figure 5**). These terms are determined via V_H and V_L alignment to consensus frameworks that provide a plane for each region about which to compare the relative orientations of the domains. The six terms include a plane-plane vector, C, four bend angles, H1 and L1, which describe V_H and V_L tilting motion toward one another, H2 and L2, which describe twisting motions between the domains, and finally, HL, which describes a torsion between the domains relative to H1 and L1 about the C vector.[3] Comparison of the orientations in the 1 μs simulations of the CH103 UCA and the CH103 UCA-P14S/S30G (**Supplementary Figure 5**) reveal HC1 and LC2 to be largely unaffected by the mutations with HC1 means of -76.8 +/- 2.1 degrees and 76.8 +/- 2.1 degrees for the UCA and UCA

P14S/S30G, respectively, and LC2 means of 80.4 +/- 3.2 degrees and 79.8 +/- 2.9 degrees for the UCA and UCA P14S/S30G, respectively. Though the dc distance histograms indicate a slight transition of the UCA P14S/S30G, the mean values for the UCA and UCA-P14S/S30G were quite similar at 15.9 +/- 0.3 Å and 15.8 +/- 0.4 Å, respectively. The LC1 distribution for the UCA-P14S/S30G is shifted toward a wider angle than that of the UCA with a mean of 117.6 +/- 2.6 degrees compared to the UCA mean of 116.4 +/- 2.4 degrees. The most dramatic shifts in orientation occur in HL and HC2. The mean values for HL were -59.8 +/- 6.2 degrees and -61.6 +/- 4.6 degrees for the UCA and $UCA_{P14S/S30G}$, respectively, with mean values for HC2 of 120.5 +/- 3.1 degrees and 118.4 +/- 2.7 degrees for the UCA and $UCA_{P14S/S30G}$, respectively. Indeed, the RMSF values for the binding site LCDR and HCDR loops are significantly higher in the mutated UCA (**Figure 5h-i**). The shifted LC1 angle is also consistent with the previous observation of a shifted V_L in the bnAb state which was shown to be important for evasion of Env V5 mutations.[4] The LC1 angle is known to be influenced predominantly by VH-VL interface core residues where mutations were found to stabilize the shifted VL orientation.[3,4]

Supplementary Figures

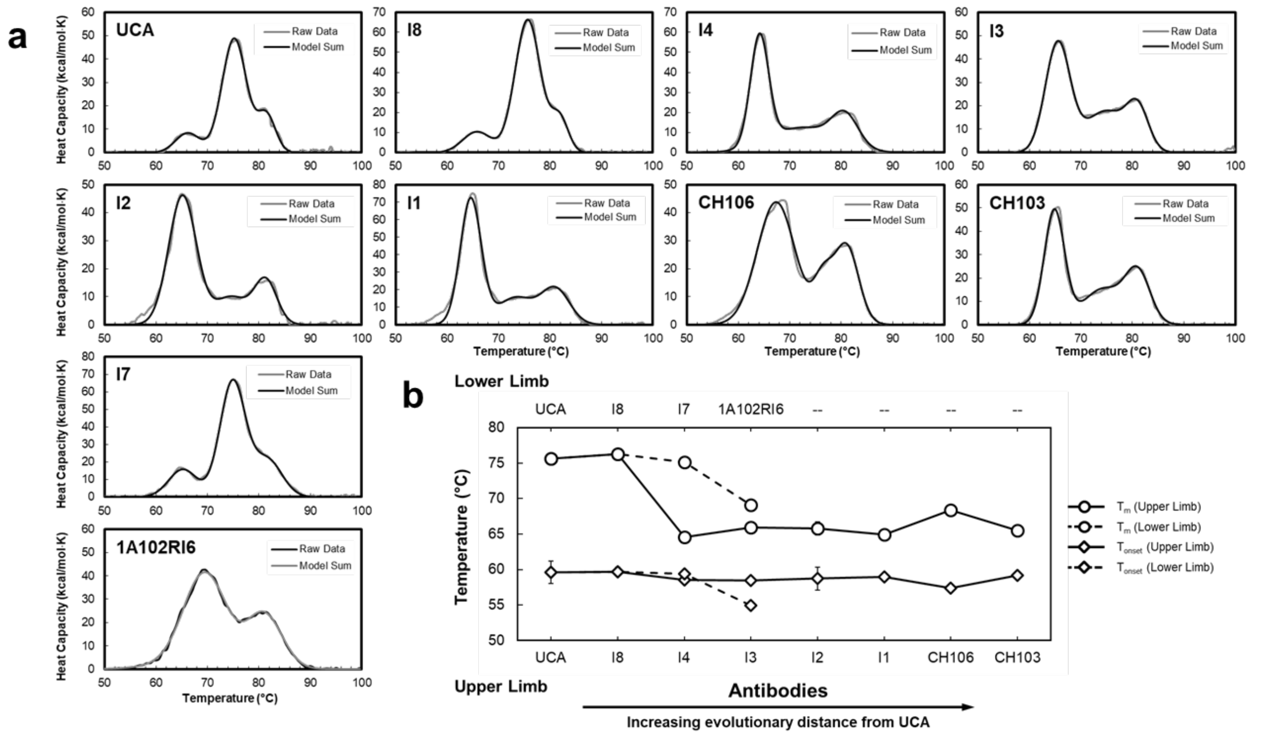

Supplementary Figure 1. Thermal Stability in the CH103 bnAb Lineage. (**a**) Antibody thermal denaturation profiles of the CH103 lineage Abs were obtained and analyzed by DSC as described in Methods. Representative raw DSC profiles (gray) are overlaid with the model sum of three Gaussian transitions (black) and demonstrate destabilization of the Fab domain (largest amplitude peak) during affinity maturation (changes in heat capacity from 20 to 50 °C were negligible and data in this range was not included). (**b**) DSC thermal denaturation analysis of the CH103 lineage mAbs of the upper limb (circle, solid line) reveals a marked decrease in Fab domain thermal stability (T_m) at the I8 to I4 transition (~10 °C) , after which, the thermal stability remains relatively constant throughout the remainder of the maturation process. The onset of whole antibody denaturation (T_{onset}; diamond, solid line) remains constant throughout the lineage. Alternatively, in the lower limb of the CH103 lineage (circle, dashed line), the T_m of I7 is comparable to the UCA with a slight decrease (6 °C) observed at the I7 to 1A102RI6 transition. This destabilization is accompanied by a concomitant decrease in T_{onset}. As we have described, destabilization during the course of antibody maturation is not uncommon, however the slight reduction in T_m observed in the lower limb, coupled with the lack of acquisition of the key mutations observed in the upper limb and lack of neutralization breadth, suggests that this destabilization is due to conformational or structural changes unique to those observed in the upper limb, the exact nature of which was not investigated further. Data are plotted as the mean and standard deviation from a minimum of two replicate measurements. Certain error bars are smaller than the data markers at this scale.

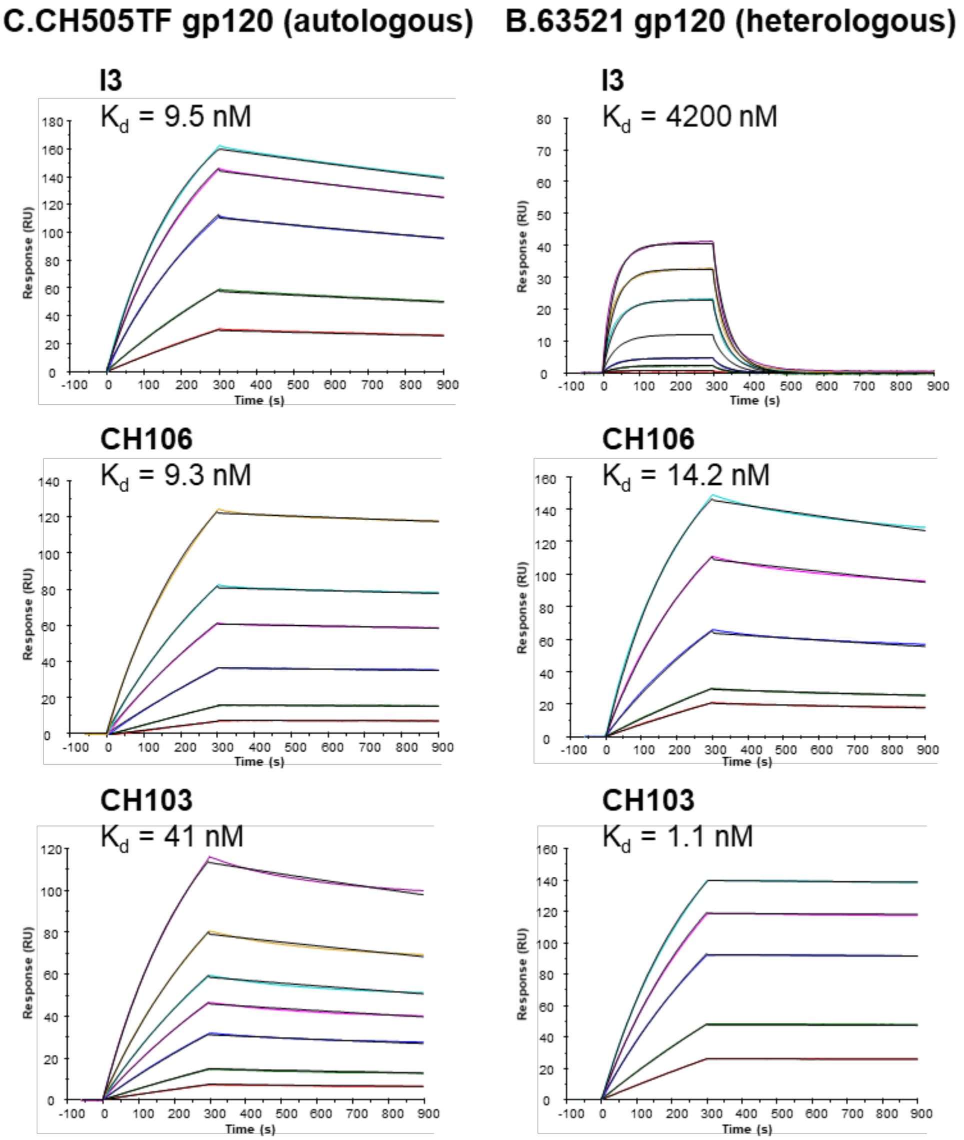

Supplementary Figure 2. SPR affinity of I3, CH106, and CH103 to C.CH505TF and B.63521 gp120. SPR kinetic titrations are shown for binding of I3 to C.CH505TF gp120 at concentrations of 20, 39, 98, 147, and 195 nM and B.63521 gp120 at concentrations of 37, 94, 187, 468, 937, 1405, and 1874 nM. SPR kinetic titrations are shown for binding of CH106 to C.CH505TF gp120 at concentrations of 20, 39, 98, 195, 293, and 489 nM and B.63521 gp120 at concentrations of 19, 37, 94, 187 and 281 nM. SPR kinetic titrations are shown for binding of CH103 to C.CH505TF gp120 at concentrations of 20, 39, 98, 147, 195, 293, 489 nM and B.63521 gp120 at concentrations of 19, 37, 94, 141, 187 nM. Binding curves are shown in color and fitted curves for each concentration are in black. Apparent affinities (K_d) obtained for each interaction are listed.

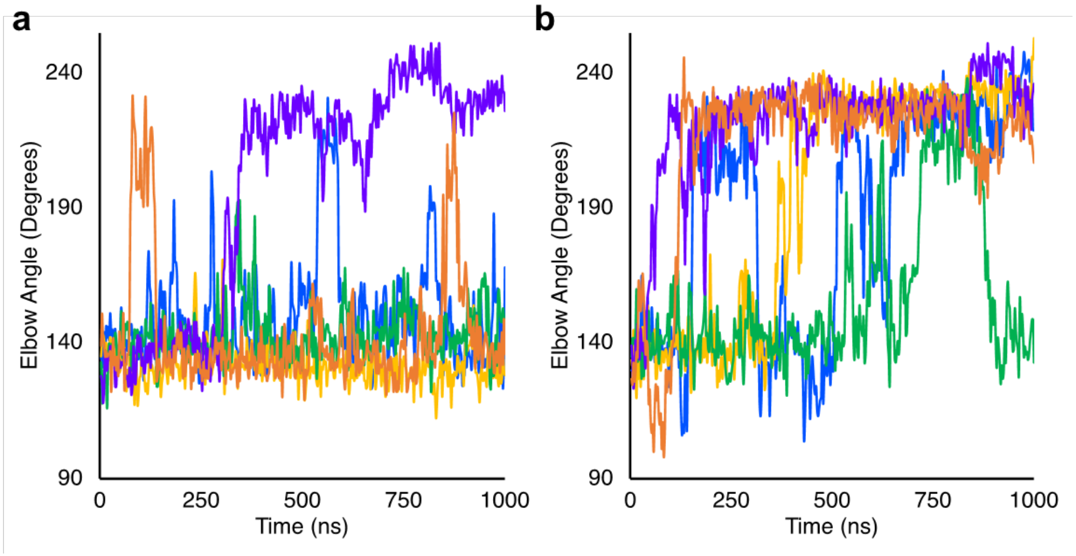

Supplementary Figure 3. CH103 UCA and P14S/S30G elbow angles during the 1 μs simulations. (**a**) Elbow angle vs. time for the five 1 μs CH103 UCA trajectories. (**b**) Elbow angle vs. time for the five 1 μs CH103 UCA P14S/S30G trajectories.

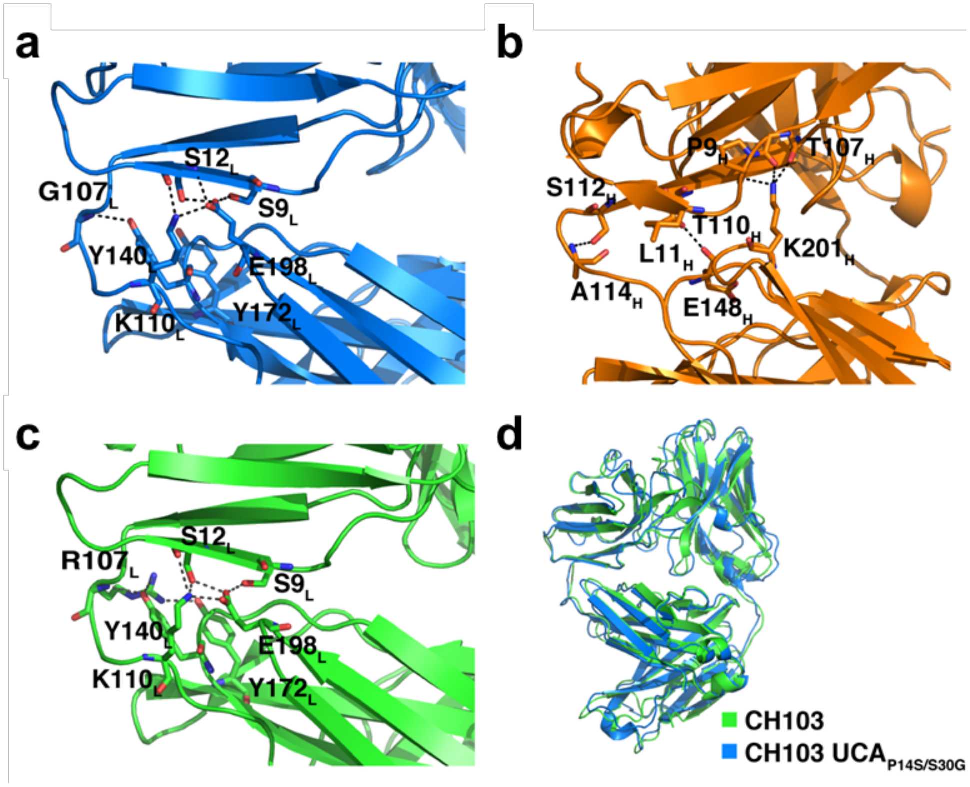

Supplementary Figure 4. Comparison of the CH103 UCA-P14S/S30G simulation to the CH103 bnAb. (**a**) Representative CH103 UCA P14S/S30G light chain, shifted position elbow joint stabilizing residue interactions. (**b**) CH103 UCA P14S/S30G heavy chain, initial position elbow joint stabilizing residue interactions. (**c**) CH103 crystal structure (PDB ID 4JAM) light chain, elbow joint stabilizing residue interactions. (**d**) Structural alignment of the CH103 UCA P14S/S30G shifted position to the CH103 crystal structure (RMSD ~1.3Å).

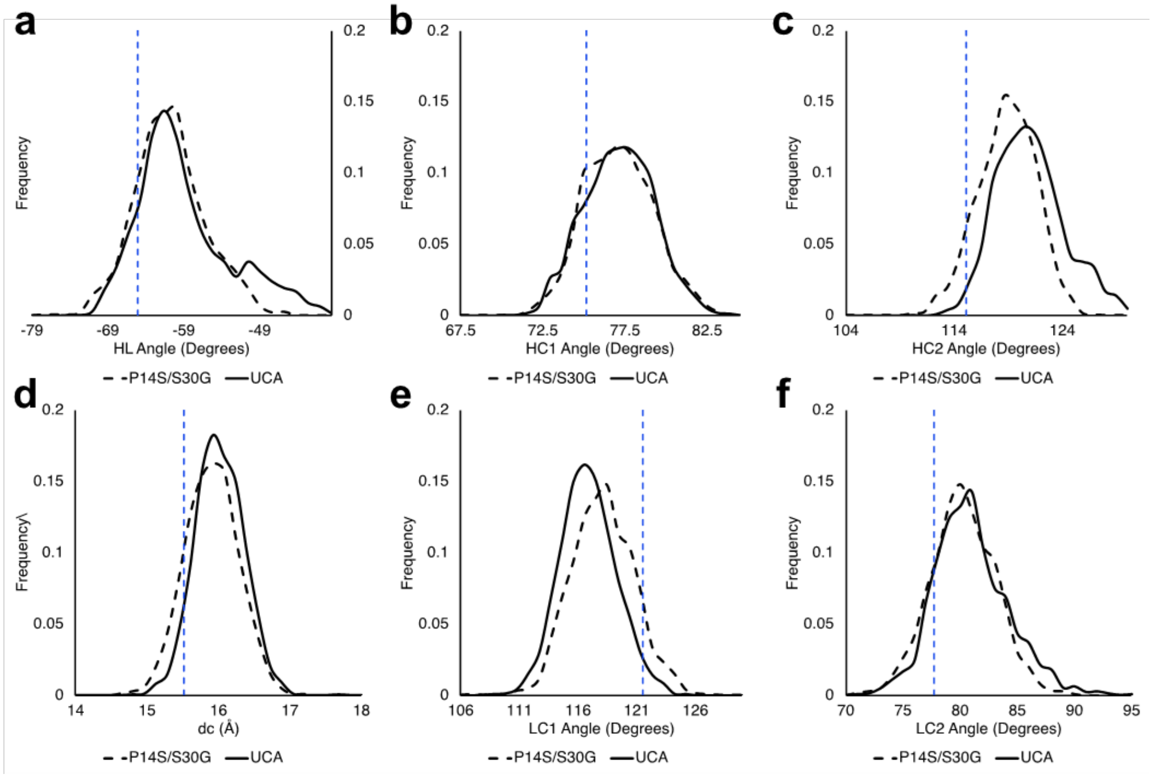

Supplementary Figure 5. CH103 ABangle V_H-V_L angle and distance distributions. (**a-f**) CH103 UCA (solid) and CH103 P14S/S30G mutant (dashed) histograms provided for the HL, HC1, LC1, HC2, LC2, and dc values. Blue dashed line indicates CH103 bnAb values (PDB ID 4JAM chains H and L).

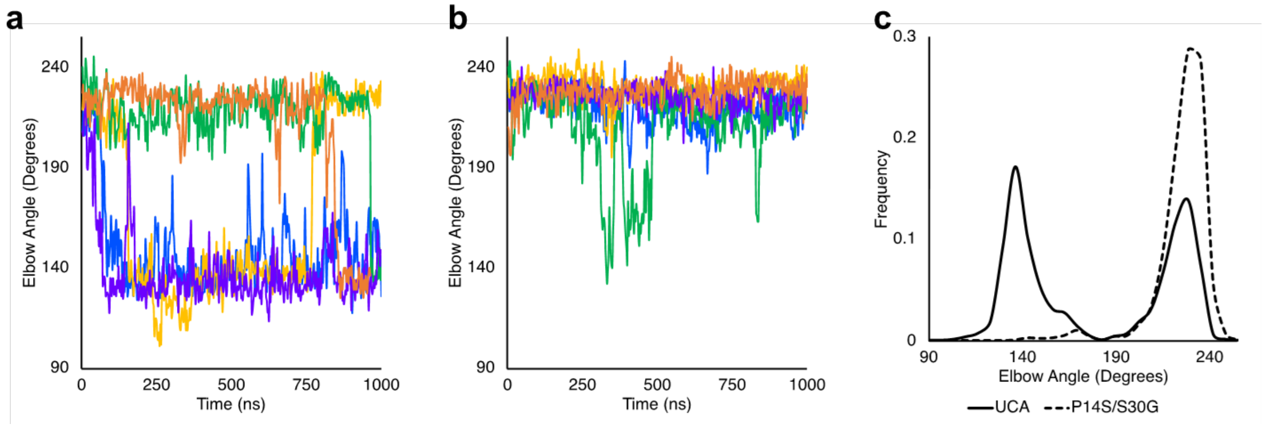

Supplementary Figure 6. CH103 UCA and UCA-P14S/S30G elbow angles during the elbow shifted 1 μs simulations. (**a**) Elbow angle vs. time for the five 1 μs CH103 UCA trajectories. (**b**) Elbow angle vs. time for the five 1 μs CH103 UCA-P14S/S30G trajectories. (**c**) Elbow angle distribution for the five 1 μs simulations of the CH103 UCA (solid) and the CH103 UCA-P14S/S30G mutant (dashed).

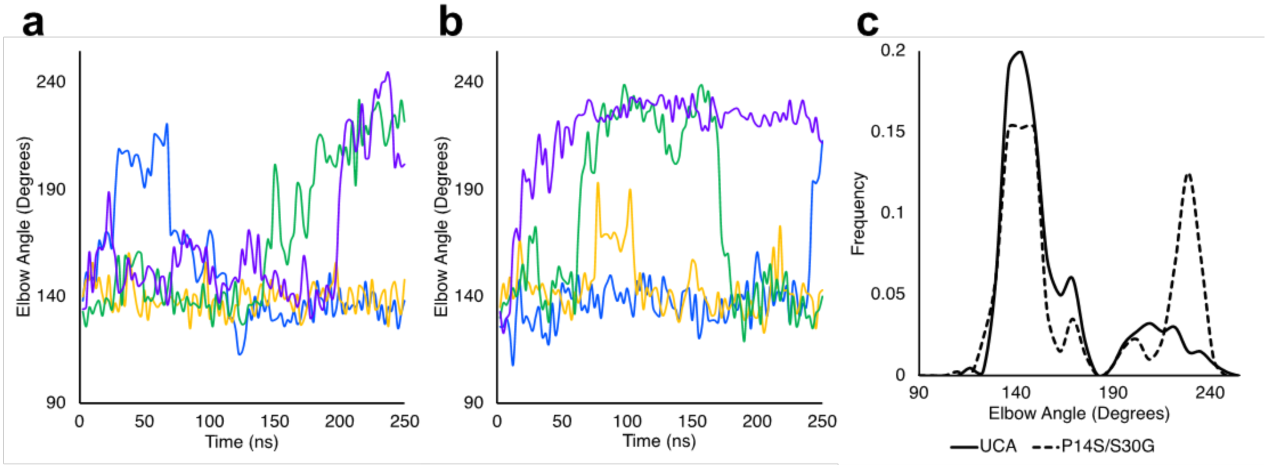

Supplementary Figure 7. CH103 UCA and P14S/S30G elbow angles during the 0.25 µs simulations. (**a**) Elbow angle vs. time for the four 0.25 µs CH103 UCA trajectories. (**b**) Elbow angle vs. time for the four 0.25 µs CH103 UCA P14S/S30G trajectories. (**c**) Elbow angle distribution for the four 0.25 µs simulations of the CH103 UCA (solid) and the CH103 UCA P14S/S30G mutant (dashed).

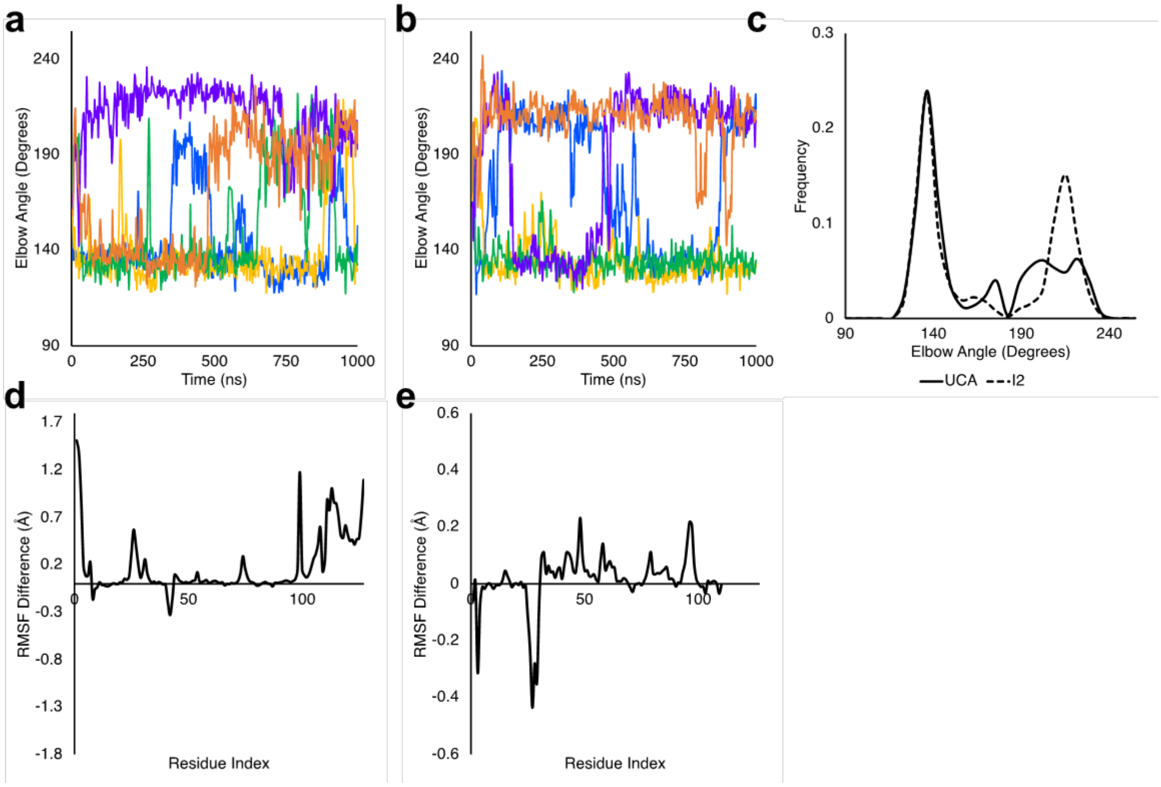

Supplementary Figure 8. DH270 1 µs simulation results. (**a**) Elbow angle vs. time for the five 1 µs DH270 UCA trajectories. (**b**) Elbow angle vs. time for the five 1 µs DH270 I2 trajectories. (**c**) Elbow angle distribution for the five 1 µs simulations of the DH270 UCA (solid) and the DH270 I2 (dashed). (**d**) V_H RMSF difference between the DH270 I2 and the DH270 UCA. (**e**) V_L RMSF difference between the DH270 I2 and the DH270 UCA.

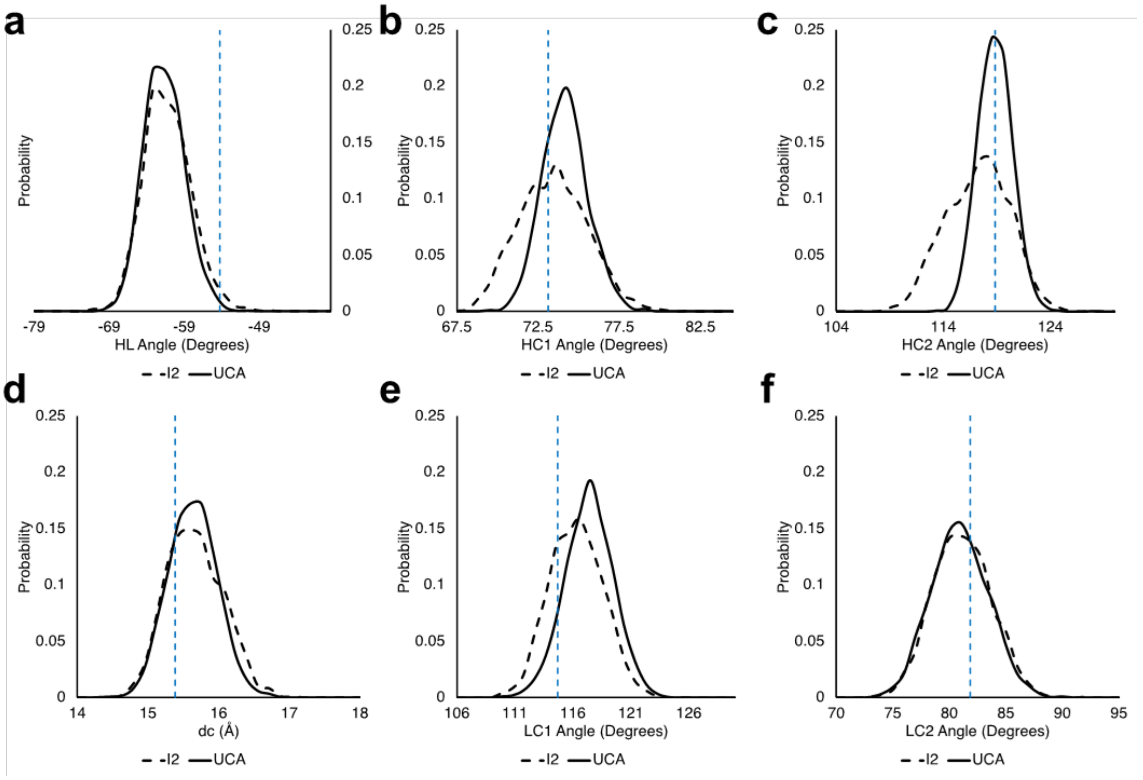

Supplementary Figure 9. DH270 ABangle V_H-V_L angle and distance distributions. (**a-f**) DH270 UCA (solid) and DH270 intermediate I2 (dashed) histograms provided for the HL, HC1, LC1, HC2, LC2, and dc values. Blue dashed line indicates DH270.6 bnAb values (PDB ID 5TQA chains A and B).

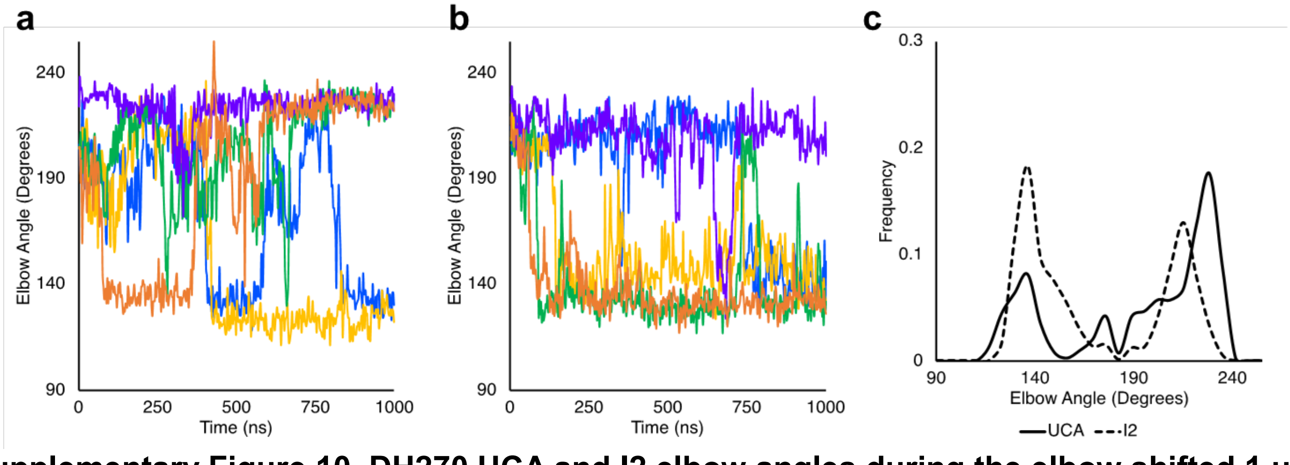

Supplementary Figure 10. DH270 UCA and I2 elbow angles during the elbow shifted 1 μs simulations. (**a**) Elbow angle vs. time for the five 1 μs DH270 UCA trajectories. (**b**) Elbow angle vs. time for the five 1 μs DH270 I2 trajectories. (**c**) Elbow angle distribution for the five 1 μs simulations of the DH270 UCA (solid) and I2 (dashed).

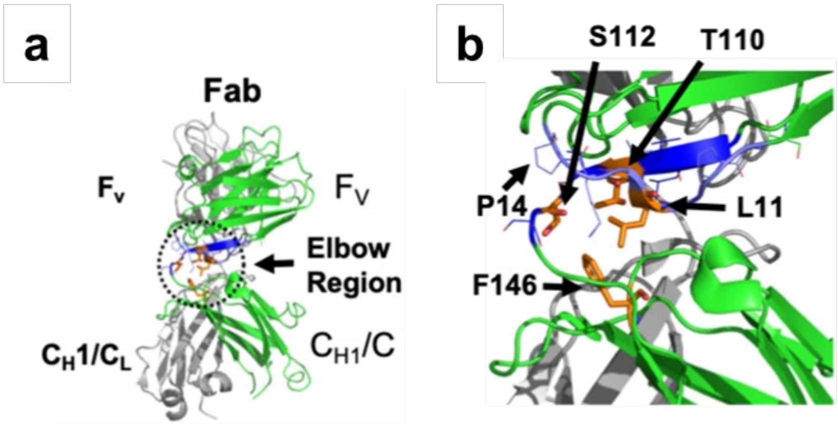

Supplementary Figure 11. BnAb elbow mutation frequencies. (**a**) Representative structure of a Fab with a formed heavy chain (green) elbow ball-and-socket (circled). (**b**) Elbow region (blue; CH103 UCA) and ball-and-socket residues (orange) of the Fab V_H used in the analysis of the elbow mutation frequency (C_{H1}/C_L F146 displayed for clarity).

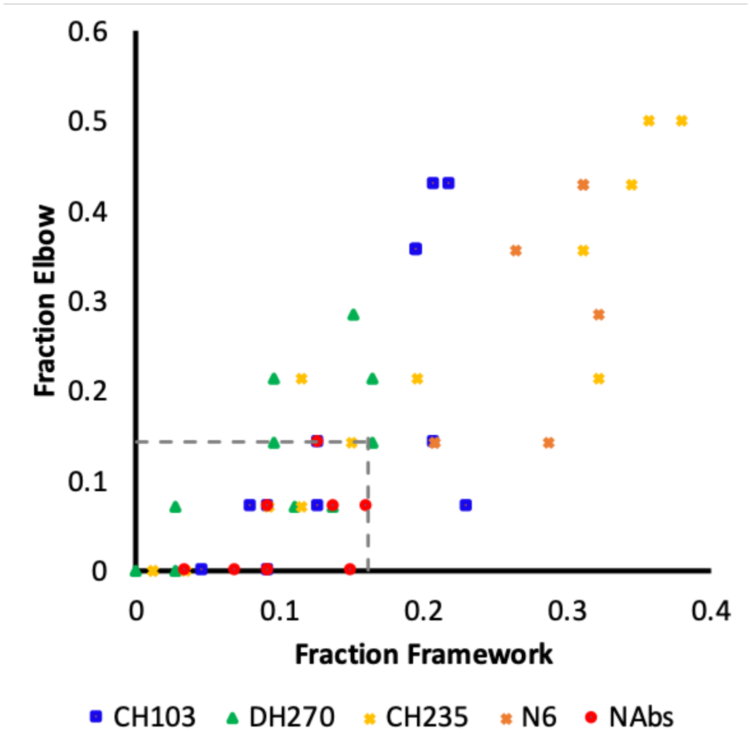

Supplementary Figure 12. Fraction elbow vs. fraction framework for Non-bnAbs and for bnAb lineages. Fraction of Fab framework mutations vs. fraction of Fab elbow mutations in the CH103, DH270, N6, and CH235 lineages as compared to that of non-bnAbs. The grey, dashed box encloses the extent of mutation in the non-bnAbs.

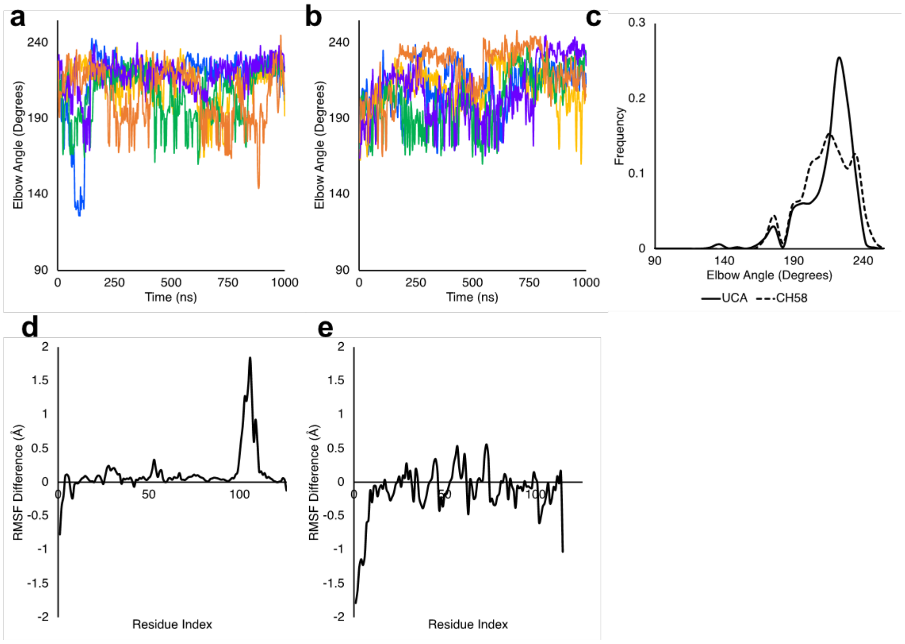

Supplementary Figure 13. CH58 1 µs simulation results. (**a**) Elbow angle vs. time for the five 1 µs CH58 UCA trajectories. (**b**) Elbow angle vs. time for the five 1 µs CH58 trajectories. (**c**) Elbow angle distribution for the five 1 µs simulations of the CH58 UCA (solid) and the CH58 (dashed). (**d**) V_H RMSF difference between CH58 and the CH58 UCA. (E) V_L RMSF difference between CH58 and the CH58 UCA.

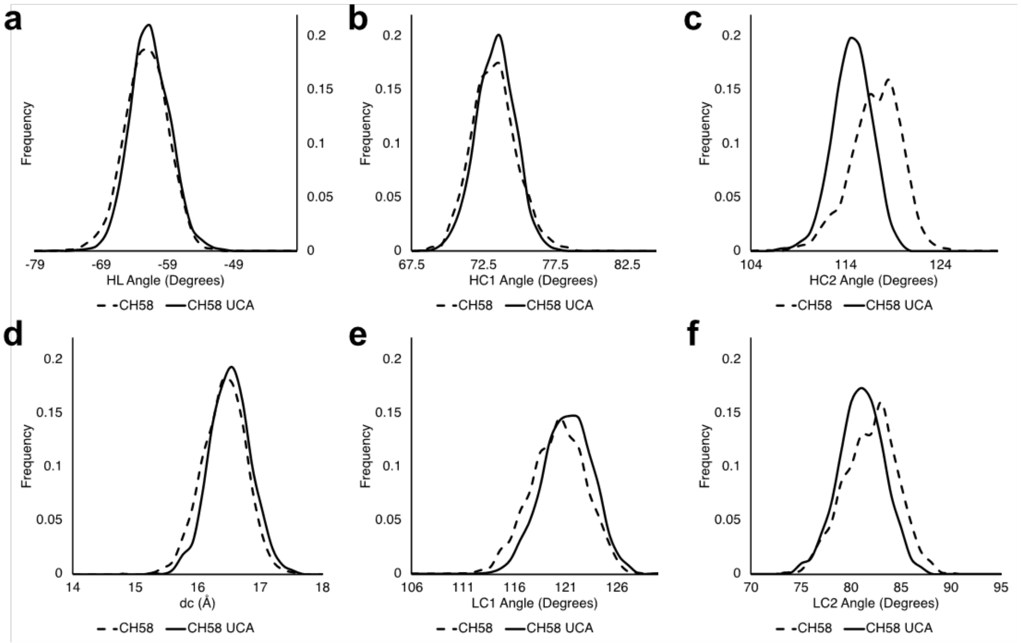

Supplementary Figure 14. CH58 ABangle V_H-V_L angle and distance distributions. (**a-f**) CH58 UCA (solid) and CH58 (dashed) histograms provided for the HL, HC1, LC1, HC2, LC2, and dc values.

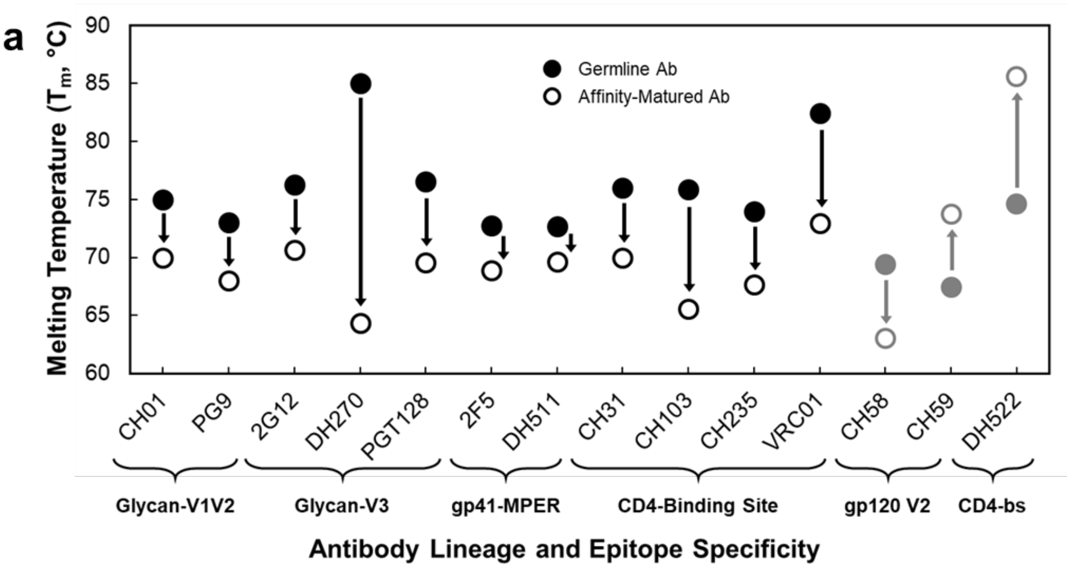

b

	DSC		DSC Model Fits		
**Non-bnAb**	**T_{onset} (°C)**	**T_m (°C)**	$T_{m,1}$ (°C)	$T_{m,2}$ (°C)	$T_{m,3}$ (°C)
CH58_UCA	58.2 ± 0.6	**69.4 ± 0.1**	65.7 ± 0.4	*69.5 ± 0.1*	73.9 ± 0.5
CH58	58.2 ± 0.03	**63.00 ± 0.03**	*62.67 ± 0.01*	74.1 ± 0.1	81.9 ± 0.1
CH59_RUA	59.2 ± 0.4	**67.4 ± 0.1**	65.62 ± 0.04	*67.50 ± 0.01*	76.4 ± 0.5
CH59	59.1 ± 0.4	**73.70 ± 0.02**	64.8 ± 0.1	*73.37 ± 0.00*	77.6 ± 0.1
DH522_UCA	63.2 ± 0.5	**74.6 ± 0.1**	72.1 ± 0.6	*74.46 ± 0.01*	79.9 ± 0.3
DH522	63.9 ± 0.6	**85.57 ± 0.05**	68.3 ± 0.2	82.6 ± 0.1	*85.41 ± 0.00*

Supplementary Figure 15. Thermal destabilization in non-bnAb affinity maturation. (**a**) Thermal denaturation of affinity-matured HIV-1 bnAbs (open circle, black), non-bnAbs (open circle, gray), and their corresponding germline or germline-proximal mAbs (closed circle) was performed by CD and/or DSC for Ab pairs of indicated epitope specificity. The germline or germline-proximal mAb demonstrated higher thermostability (T_m) than the affinity-matured mAb for all classes of bnAb and for CH58. Alternatively, the germline mAb for the gp120 V2 non-bnAb CH59 and the CH505 Env induced rhesus CD4-binding site mAb DH522[5] had lower thermostabilities than their corresponding affinity-matured mAbs. (**b**) Antibody thermal denaturation profiles were obtained and analyzed by DSC as described in Methods. The raw data was best fit with three Gaussian transition models ($T_{m,1\text{-}3}$). The largest amplitude model (indicated in italics) corresponds to the denaturation of the Fab domain and is in good agreement with the observed primary T_m. Data are plotted as the mean and standard deviation from a minimum of two replicate measurements. Certain error bars are smaller than the data markers at this scale. Data shown for CH01, PG9, and CH31 Ab pairs are the result of single measurements by CD with replicate measurements by DSC reported in **Supplementary Table 1**.

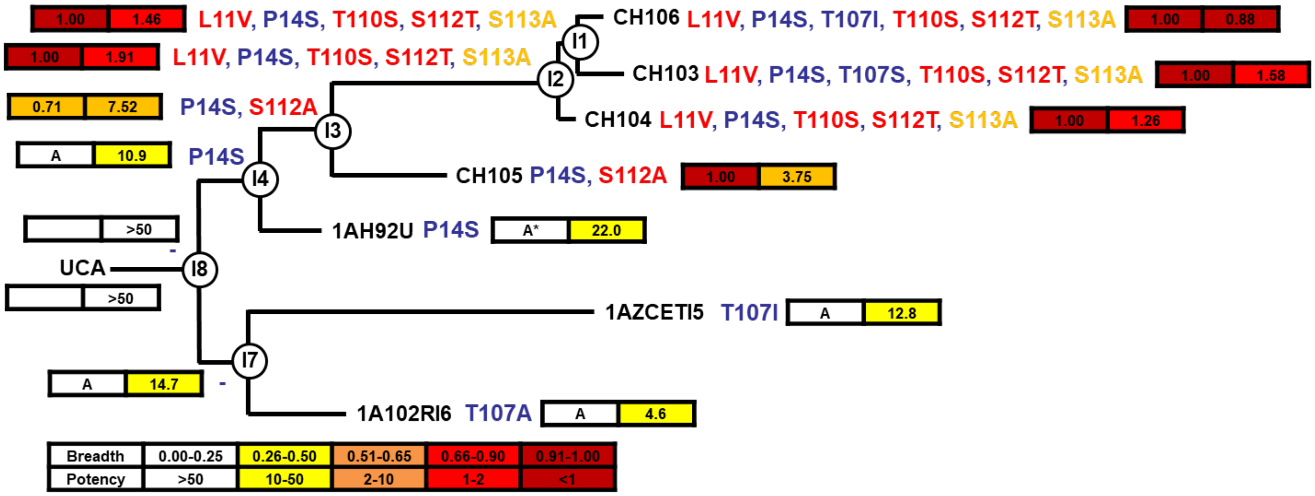

Supplementary Figure 16. CH103 lineage elbow angle mutations vs. neutralization breadth and potency. CH103 lineage elbow angle mutations in the V_H elbow ball-and-socket residues (red), residues adjacent to ball-and-socket (yellow), and residues in the elbow region (blue); Boxes depict the neutralization breadth (left) and the geometric mean potency determined previously (right; IC_{50}, µg/ml, a value of 50 was used for those with IC_{50} > 50). An "A" for the neutralization breadth indicates the antibody neutralizes autologous virus only. A* for 1AH92U indicates this antibody did not neutralize autologous virus. The potency value listed is for the only virus neutralized, B.SF162. The V_HDJ_H genes of I1–I4, I7 and I8 were paired with either the V_L gene of the inferred UCA or I2 antibodies, and the mature CH103 was paired with its natural gene as described earlier.[6] Neutralization data are from previously published work.[6]

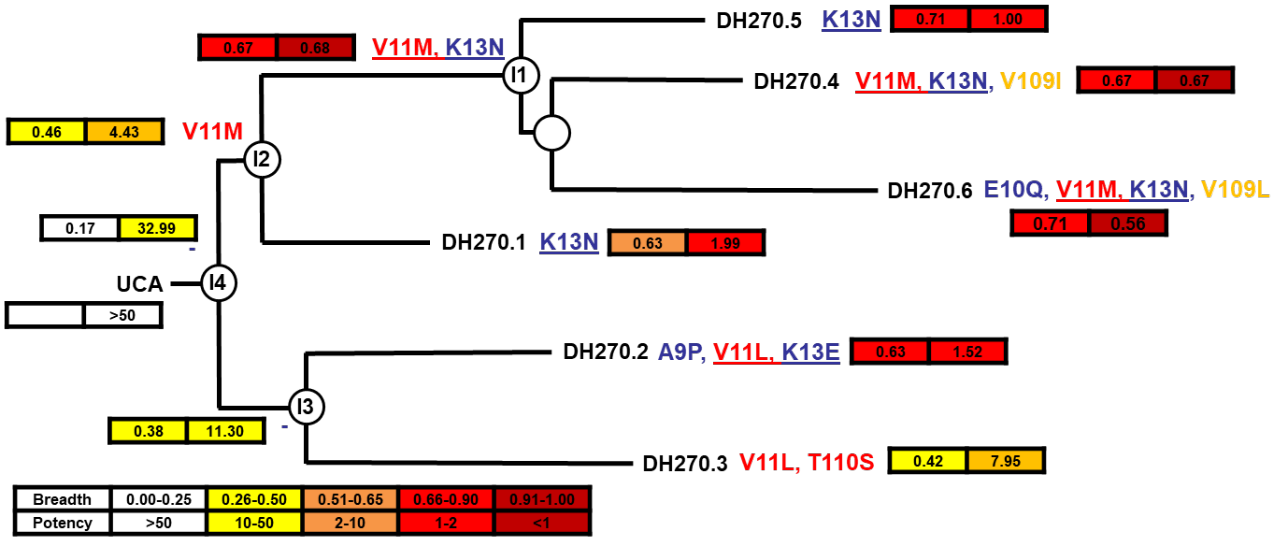

Supplementary Figure 17. DH270 lineage elbow angle mutations vs. neutralization breadth and potency. V_H elbow ball-and-socket residues (red), residues adjacent to ball-and-socket (yellow), and residues in the elbow region (blue); Boxes depict the neutralization breadth (left) and the geometric mean (IC_{50}, µg/ml) potency (right; a value of 50 was used for those with IC_{50} > 50). Neutralization data are from previously published work.[7] Intermediate (I) antibodies are referenced as IA in Bonsignori et al.

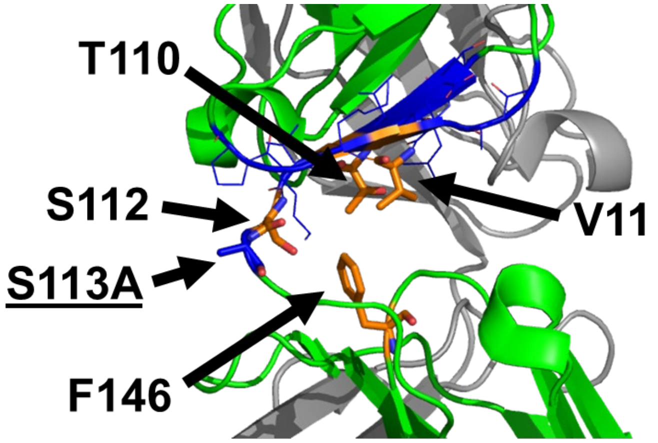

Supplementary Figure 18. CH235 V_H elbow joint residues. CH235 V_H elbow region (blue) and ball-and-socket (orange) residues identifying the single CH235 elbow joint mutation relative to the CH235 UCA (underlined).

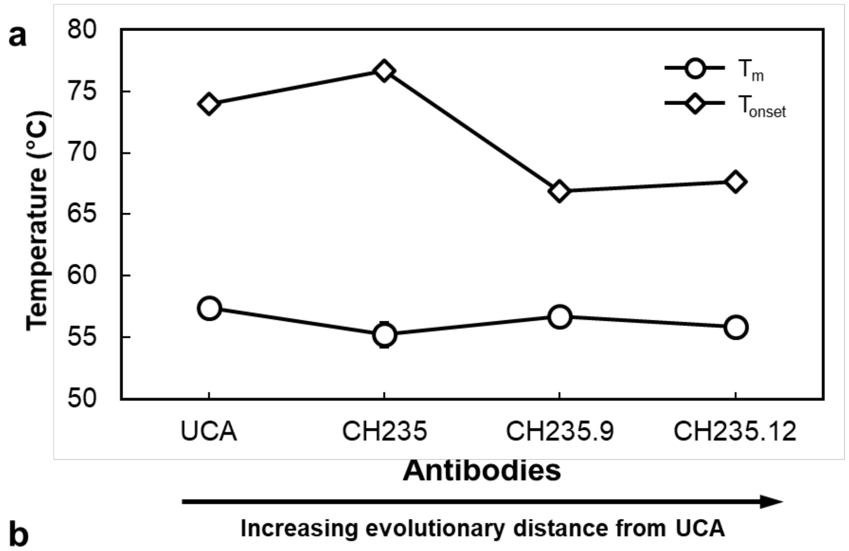

b

	DSC		DSC Model Fits		
**CH235 mAb**	**T_{onset} (°C)**	**T_m (°C)**	$T_{m,1}$ (°C)	$T_{m,2}$ (°C)	$T_{m,3}$ (°C)
UCA	57.4 ± 0.5	**73.96 ± 0.01**	65.4 ± 0.3	*73.74 ± 0.04*	80.8 ± 0.2
CH235	55 ± 1	**76.65 ± 0.01**	65.1 ± 0.7	*76.21 ± 0.04*	82.85 ± 0.03
CH235.9	56.8 ± 0.2	**66.9 ± 0.1**	*66.6 ± 0.1*	71.9 ± 0.1	82.5 ± 0.1
CH235.12	55.9 ± 0.8	**67.7 ± 0.1**	*67.2 ± 0.1*	70.4 ± 0.4	82.53 ± 0.04

Supplementary Figure 19. DSC Thermal Denaturation of the CH235 bnAb Lineage. (**a**) Thermal denaturation of select antibodies of the CH235 lineage was measured by differential scanning calorimetry at a scan rate of 1 °C/min from 20 to 100 °C. Destabilization was observed at the CH235 to CH235.9 transition from 74-76 °C for UCA and CH235 to 67 °C for CH235.9 and CH2345.12, while the transition onset temperatures (T_{onset}) remained comparable throughout the lineage. (**b**) Antibody thermal denaturation profiles were obtained and analyzed by DSC as described in Methods. The raw data was best fit with three Gaussian transition models ($T_{m,1-3}$). The largest amplitude model (indicated in italics) corresponds to the denaturation of the Fab domain and is in good agreement with the observed primary T_m. Data are plotted as the mean and standard deviation from a minimum of two replicate measurements. Certain error bars are smaller than the data markers at this scale.

Supplementary Tables

Supplementary Table 1. Thermal denaturation of affinity matured HIV-1 bnAbs and corresponding germline or germline-proximal mAbs.

	DSC		DSC Model Fits			CD
**mAb**	**T_{onset} (°C)**	**T_m (°C)**	$T_{m,1}$ (°C)	$T_{m,2}$ (°C)	$T_{m,3}$ (°C)	**T_m (°C)**
CH01_RUA	58 ± 1	**79.190 ± 0.002**	64.2 ± 0.1	77.5 ± 0.1	*79.27 ± 0.02*	75.0
CH01	64.91 ± 0.02	**72.11 ± 0.01**	*71.54 ± 0.01*	78.8 ± 0.3	83.3 ± 0.2	70.0
PG9_RUA	65.5 ± 0.1	**72.7 ± 0.1**	70.9 ± 0.2	*72.72 ± 0.01*	83.60 ± 0.01	73.0
PG9	55.8 ± 0.2	**62.09 ± 0.01**	61.50 ± 0.04	*61.86 ± 0.02*	75.98 ± 0.00	68.0
2G12_UCA	64.9 ± 0.2	**76.29 ± 0.02**	74.0 ± 0.2	*76.9 ± 0.1*	83.91 ± 0.04	ND
2G12	55.23 ± 0.03	**70.6 ± 0.2**	*68.90 ± 0.04*	71.4 ± 0.1	84.03 ± 0.01	ND
DH270_UCA4	58.9 ± 0.2	**85.0 ± 0.1**	65.00 ± 0.05	81.68 ± 0.05	*85.03 ± 0.01*	ND
DH270.6	59.3 ± 0.3	**64.34 ± 0.01**	63.27 ± 0.04	*64.26 ± 0.04*	78.2 ± 0.2	ND
PGT128_UCA	54.4 ± 0.5	**76.54 ± 0.01**	65.3 ± 0.4	*76.01 ± 0.01*	81.4 ± 0.1	ND
PGT128	64.3 ± 0.4	**69.56 ± 0.03**	*69.23 ± 0.02*	77.1 ± 0.1	84.22 ± 0.01	ND
2F5_RUA-N	61.9 ± 0.8	**72.73 ± 0.03**	67.3 ± 0.2	*72.27 ± 0.01*	82.6 ± 0.5	71 ± 1
2F5	59.1 ± 0.2	**68.9 ± 0.1**	*68.35 ± 0.01*	70.5 ± 0.4	83.8 ± 0.1	69.0
DH511_I6	58.3 ± 0.3	**72.71 ± 0.01**	68 ± 1	*72.5 ± 0.1*	80.9 ± 0.3	ND
DH512	58.0 ± 0.1	**69.7 ± 0.3**	*67.5 ± 0.1*	73.4 ± 0.3	81.62 ± 0.05	ND
CH31_UCA	65.3 ± 0.2	**85.53 ± 0.01**	71.66 ± 0.01	83.5 ± 0.1	*85.8 ± 0.1*	76.0
CH31	57.8 ± 0.1	**65.7 ± 0.1**	*65.56 ± 0.00*	71.62 ± 0.04	80.6 ± 0.3	70.0
CH103_UCA	59.6 ± 1.6	**75.6 ± 0.3**	66 ± 1	*75.0 ± 0.3*	81.3 ± 0.1	75.98 ± 0.03
CH103	59.2 ± 0.1	**65.5 ± 0.1**	*64.85 ± 0.02*	74.7 ± 0.5	81.3 ± 0.3	69.0
CH235_UCA	57.4 ± 0.5	**73.96 ± 0.01**	65.4 ± 0.3	*73.74 ± 0.04*	80.8 ± 0.2	ND
CH235.12	55.9 ± 0.8	**67.7 ± 0.1**	*67.2 ± 0.1*	70.4 ± 0.4	82.53 ± 0.04	ND
VRC01_UCA	56.4 ± 0.4	**82.46 ± 0.02**	64.7 ± 0.4	80.2 ± 0.1	*82.50 ± 0.01*	ND
VRC01	63.1 ± 0.5	**72.93 ± 0.01**	70.8 ± 0.3	*72.91 ± 0.04*	82.7 ± 0.1	ND

Antibody thermal denaturation profiles were obtained and analyzed by DSC and CD as described in Methods. The DSC raw data was best fit with three Gaussian transition models ($T_{m,1\text{-}3}$). The largest amplitude model (indicated in italics) corresponds to the denaturation of the Fab domain and is in good agreement with the observed primary T_m. Data are shown as the mean and standard deviation from a minimum of two replicate measurements unless listed as a single value. ND = Not determined.

Supplementary Table 2. DSC Thermal denaturation of the CH103 lineage mutants.

	**DSC**		DSC Model Fits		
**CH103 mAb**	**T_{onset} (°C)**	**T_m (°C)**	$T_{m,1}$ (°C)	$T_{m,2}$ (°C)	$T_{m,3}$ (°C)
UCA	60 ± 2	**75.6 ± 0.3**	66 ± 1	*75.0 ± 0.3*	81.3 ± 0.1
UCA-P14S	57 ± 1	**74.6 ± 0.1**	65.62 ± 0.03	*74.1 ± 0.01*	80.6 ± 0.1
UCA-S30G	57.7 ± 0.3	**74.6 ± 0.1**	65.6 ± 0.1	*74.08 ± 0.01*	80.73 ± 0.04
UCA-S30G/S31G	58 ± 2	**73.8 ± 0.4**	65.2 ± 0.3	*73.48 ± 0.01*	80.51 ± 0.05
I4	58.6 ± 0.4	**64.60 ± 0.01**	*64.13 ± 0.01*	74 ± 3	81.0 ± 0.4
I4-S14P	58.4 ± 0.2	**67.01 ± 0.04**	*66.80 ± 0.04*	75.7 ± 0.4	81.1 ± 0.1
I4-S14P/G30S	58.7 ± 0.7	**68.42 ± 0.02**	*68.07 ± 0.04*	75.8 ± 0.1	81.0 ± 0.1
CH103	59.2 ± 0.1	**65.5 ± 0.1**	*64.85 ± 0.02*	74.7 ± 0.5	81.3 ± 0.3
CH103-S14P	59.1 ± 0.2	**66.41 ± 0.03**	*66.24 ± 0.01*	77 ± 1	81.9 ± 0.4
CH103-S14P/G30S	59.47 ± 0.01	**66.57 ± 0.05**	*67.02 ± 0.04*	75.9 ± 0.9	81.6 ± 0.3

Antibody thermal denaturation profiles were obtained and analyzed by DSC as described in Methods. The raw data was best fit with three Gaussian transition models ($T_{m,1\text{-}3}$). The largest amplitude model (indicated in italics) corresponds to the denaturation of the Fab domain and is in good agreement with the observed primary T_m. Data are shown as the mean and standard deviation from a minimum of two replicate measurements unless listed as a single value. ND = Not determined.

Supplementary Table 3. Kinetics and apparent affinity of the CH103 lineage mutants for transmitted founder (TF) and heterologous HIV-1 Env.

	C.CH505TF gp120			C.CH505TF.SOSIP.664.v4.1			B.63521 D11 gp120		
**CH103 mAb**	**k_a ($M^{-1}s^{-1}$) x 10^4**	**k_d (s^{-1}) x 10^{-4}**	**K_d (nM)**	**k_a ($M^{-1}s^{-1}$) x 10^4**	**k_d (s^{-1}) x 10^{-4}**	**K_d (nM)**	**k_a ($M^{-1}s^{-1}$) x 10^4**	**k_d (s^{-1}) x 10^{-4}**	**K_d (nM)**
UCA	2.35 ± 0.07	101 ± 6	430 ± 20	0.11 ± 0.02	16.8 ± 0.2	1500 ± 300	NB	NB	NB
UCA-P14S	3.0 ± 0.2	112 ± 3	370 ± 30	0.09 ± 0.02	15 ± 1	1700 ± 400	NB	NB	NB
UCA-S30G	2.96 ± 0.06	117 ± 3	400 ± 10	0.086 ± 0.001	15 ± 1	1800 ± 100	NB	NB	NB
UCA-S30G/S31G	2.52 ± 0.08	110 ± 2	440 ± 20	0.14 ± 0.02	12 ± 1	800 ± 100	NB	NB	NB
I4	1.2 ± 0.1	6.4 ± 0.4	53 ± 5	0.21 ± 0.07	1.3 ± 0.2	60 ± 20	0.27 ± 0.04	390 ± 50	14000 ± 3000
I4-S14P	1.31 ± 0.09	5.3 ± 0.1	40 ± 3	0.246 ± 0.001	2.7 ± 0.1	109 ± 6	ND	ND	ND
I4-S14P/G30S	1.0 ± 0.2	10.1 ± 0.8	100 ± 20	0.212 ± 0.006	3.4 ± 0.2	160 ± 10	ND	ND	ND
CH103	0.6 ± 0.2	2.6 ± 0.2	41 ± 9	0.093 ± 0.003	0.15 ± 0.03	16 ± 3	1.5 ± 0.7	0.2 ± 0.1	1.1 ± 1.0
CH103-S14P	0.52 ± 0.06	2.62 ± 0.09	50 ± 6	0.089 ± 0.002	0.18 ± 0.05	20 ± 5	0.91 ± 0.02	0.20 ± 0.04	2.1 ± 0.4
CH103-S14P/G30S	0.4 ± 0.1	3.0 ± 0.1	70 ± 20	0.045 ± 0.008	0.66 ± 0.07	150 ± 30	0.821 ± 0.003	0.29 ± 0.09	4 ± 1

Association and dissociation rate constants (k_a, k_d) were measured by SPR analysis for autologous C.CH505TF gp120 and heterologous B.63521 D11 gp120 and by BLI analysis for autologous C.CH505TF.SOSIP.664.v4.1 as described in Methods and used to calculate the apparent dissociation constant (K_d) for each mAb. Data are shown as the mean and standard deviation from a minimum of two replicate measurements. NB = no binding, ND = not determined.

Supplementary Table 4. Thermal Denaturation of the CH103 bnAb Lineage.

	DSC		DSC Model Fits			CD
**CH103 mAb**	**T_{onset} (°C)**	**T_m (°C)**	$T_{m,1}$ (°C)	$T_{m,2}$ (°C)	$T_{m,3}$ (°C)	**T_m (°C)**
UCA	60 ± 2	**75.6 ± 0.3**	66 ± 1	*75.0 ± 0.3*	81.3 ± 0.1	75.98 ± 0.03
I8	59.7 ± 0.6	**76.25 ± 0.01**	65.7 ± 0.1	*75.64 ± 0.00*	81.81 ± 0.01	76.0
I4	58.6 ± 0.4	**64.60 ± 0.01**	*64.13 ± 0.01*	74 ± 3	81.0 ± 0.4	67.0
I3	58.4 ± 0.4	**65.9 ± 0.1**	*65.4 ± 0.1*	74.7 ± 0.1	81.2 ± 0.2	68 ± 4
I2	59 ± 2	**65.8 ± 0.9**	*65.8 ± 0.9*	74.5 ± 0.1	81.2 ± 0.1	67 ± 3
I1	59.0 ± 0.2	**64.91 ± 0.02**	*64.58 ± 0.04*	73.7 ± 0.6	81.2 ± 0.3	66.0
CH106	57.4 ± 0.4	**68.4 ± 0.3**	*67.5 ± 0.1*	78 ± 2	81.8 ± 0.7	63.98 ± 0.00
CH103	59.2 ± 0.1	**65.5 ± 0.1**	*64.85 ± 0.02*	74.7 ± 0.5	81.3 ± 0.3	69.0
I7	59.4 ± 0.5	**75.09 ± 0.01**	65.1 ± 0.2	*74.89 ± 0.01*	81.63 ± 0.04	72.0
1A102RI6	55.0 ± 0.5	**69.1 ± 0.6**	62 ± 2	*69.51 ± 0.02*	80.9 ± 0.1	ND

Antibody thermal denaturation profiles were obtained and analyzed by DSC and CD as described in Methods. The DSC raw data was best fit with three Gaussian transition models ($T_{m,1\text{-}3}$). The largest amplitude model (indicated in italics) corresponds to the denaturation of the Fab domain and is in good agreement with the observed primary T_m. Data are shown as the mean and standard deviation from a minimum of two replicate measurements unless listed as a single value. ND = Not determined.

Supplementary Table 5. Kinetics and apparent affinity of the CH103 lineage mAbs for transmitted founder (TF) and heterologous HIV-1 Env.

	C.CH505TF gp120			C.CH505TF.SOSIP.664.v4.1			B.63521 D11 gp120		
**CH103 mAb**	**k_a ($M^{-1}s^{-1}$) x 10^4**	**k_d (s^{-1}) x 10^{-4}**	**K_d (nM)**	**k_a ($M^{-1}s^{-1}$) x 10^4**	**k_d (s^{-1}) x 10^{-4}**	**K_d (nM)**	**k_a ($M^{-1}s^{-1}$) x 10^4**	**k_d (s^{-1}) x 10^{-4}**	**K_d (nM)**
UCA	2.35 ± 0.07	101 ± 6	430 ± 20	0.11 ± 0.02	16.8 ± 0.2	1500 ± 300	NB	NB	NB
I8	ND	ND	ND	ND	ND	ND	0.017	1130	680000
I4	1.2 ± 0.1	6.4 ± 0.4	53 ± 5	0.21 ± 0.07	1.3 ± 0.2	60 ± 20	0.27 ± 0.04	390 ± 50	14000 ± 3000
I3	2.45 ± 0.01	2.335 ± 0.007	9.53 ± 0.06	ND	ND	ND	0.53 ± 0.02	225 ± 3	4200 ± 100
CH106	0.75 ± 0.05	0.70 ± 0.05	9.3 ± 0.8	0.067 ± 0.002	0.036 ± 0.006	5 ± 1	1.6 ± 0.1	2.29 ± 0.02	14.2 ± 0.9
CH103	0.6 ± 0.2	2.6 ± 0.2	40 ± 10	0.093 ± 0.003	0.15 ± 0.03	16 ± 3	1.5 ± 0.7	0.2 ± 0.1	1.1 ± 1.0
I7	1.16	4.95	42.7	ND	ND	ND	0.3 ± 0.3	450 ± 20	20000 ± 20000
1A102RI6	1.13	7.44	65.8	ND	ND	ND	0.3 ± 0.1	400 ± 70	15000 ± 8000
1AZCETI5	1.14	2.83	24.8	ND	ND	ND	0.3 ± 0.03	53 ± 0.9	1800 ± 200

Association and dissociation rate constants (k_a, k_d) were measured by SPR analysis for autologous C.CH505TF gp120 and heterologous B.63521 D11 gp120 and by BLI analysis for autologous C.CH505TF.SOSIP.664.v4.1 as described in Methods and used to calculate the apparent dissociation constant (K_d) for each mAb. Data are shown as the mean and standard deviation from a minimum of two replicate measurements. NB = no binding, ND = not determined.

Supplementary Table 6. DSC thermal denaturation of the DH270 bnAb lineage.

	DSC		DSC Model Fits		
**DH270 mAb**	**T_{onset} (°C)**	**T_m (°C)**	$T_{m,1}$ (°C)	$T_{m,2}$ (°C)	$T_{m,3}$ (°C)
UCA	58.9 ± 0.2	**85.0 ± 0.1**	65.00 ± 0.05	81.68 ± 0.05	*85.03 ± 0.01*
I4	58.8 ± 0.4	**85.72 ± 0.01**	64.8 ± 0.2	82.2 ± 0.1	*85.52 ± 0.03*
I2	60.4 ± 0.4	**72.3 ± 0.1**	66.1 ± 0.3	*71.91 ± 0.01*	81.1 ± 0.3
DH270.1	58.2 ± 0.2	**66.8 ± 0.2**	*66.20 ± 0.00*	74.13 ± 0.03	80.45 ± 0.05
I1	57.4 ± 0.1	**62.7 ± 0.1**	*61 ± 1*	62.54 ± 0.02	78.51 ± 0.01
DH270.4	53.5 ± 0.1	**60.0 ± 0.2**	*59.7 ± 0.1*	61.8 ± 0.1	78.45 ± 0.02
DH270.6	59.3 ± 0.3	**64.34 ± 0.01**	*63.27 ± 0.04*	64.26 ± 0.04	78.2 ± 0.2
I3	56.4 ± 0.3	**77.48 ± 0.01**	64.15 ± 0.04	*77.21 ± 0.00*	83.3 ± 0.2
DH270.2	53.7 ± 0.1	**59.25 ± 0.02**	*58.96 ± 0.01*	71.3 ± 0.2	81.0 ± 0.4

Antibody thermal denaturation profiles were obtained and analyzed by DSC as described in Methods. The raw data was best fit with three Gaussian transition models ($T_{m,1\text{-}3}$). The largest amplitude model (indicated in italics) corresponds to the denaturation of the Fab domain and is in good agreement with the observed primary T_m. Data are shown as the mean and standard deviation from a minimum of two replicate measurements.

Supplementary Table 7. Light chain variable domain elbow region mutations

bnAb	Epitope	V_L Elbow Mutations	V_L
**8ANC131**	CD4 BS	G9A, I106R	2
**CH103**	CD4 BS	-	0
**N6**	CD4 BS	A13V, V15I, E105H	3
**VRC01**	CD4 BS	E105Q, I106V, K107D	3
**CH235.12**	CD4 BS	V13A, E105D	2
**VRC-CH31**	CD4 BS	V15L, I83V, E105D	3
**1B2530**	CD4 BS	T14A	1
**3BNC117**	CD4 BS	I106L	1
**8ANC195**	gp120-gp41	V15I, I106V	2
**35O22**	gp120-gp41	P15L, T105S	2
**PGT151**	gp120-gp41	E105D, I106L	2
**VRC34**	gp41	-	0
**DH511.2**	MPER	L11V, E105D, K107R	3
**2F5**	MPER	E105D, I106V, K107R	3
**4E10_Fab**	MPER	L11Q, I106V	2
**10E8_Fab**	MPER	P8T, A9G, E83D	3
**PGDM1400**	V1/V2	L9H, P12S, E105D	3
**PGT145**	V1/V2	S10F	1
**PG9**	V1/V2	-	0
**CH01**	V2	-	0
**BF520_1_VH**	V3	P15L, D105H	2
**DH270**	V3	L107I	1
**DH270.6**	V3	-	0
**PGT128**	V3	-	0
**PGT135**	V3	A9D, F83V, E105D	3
**PGT121**	V3	P8S, S9D, V11I	3

Supplementary Table 8. Non-bnAb elbow region mutation frequencies.

non-bnAb	Epitope	% FWR	% Elbow	V_H Elbow Mutations	V_H***	V_H*	V_H
**11-989**	CD4 BS	6.9	0.0	-	0	0	0
**6-187**	CD4 BS	9.2	0.0	-	0	0	0
**9-913**	CD4 BS	12.6	14.3	K12G*,T107S	0	1	2
**1-479**	CD4 BS/CD4i	14.9	0.0	-	0	0	0
**2-491**	CD4 BS/CD4i	9.2	7.1	T107N	0	0	1
**17b**	CD4i	13.8	7.1	S112T***	1	0	1
**1-591**	gp120 core VL	9.2	0.0	-	0	0	0
**10-188**	Glycan V3	16.1	7.1	G10T*	0	1	1
**447-52D**	V3	3.4	0.0	-	0	0	0
**CH58**	V2	1.1	0.0	-	0	0	0
**CH59**	V2	2.3	0.0	-	0	0	0

V_H framework and elbow region mutation frequencies (%) are relative to the unmutated common ancestor. Asterisks indicate whether residues occur in the ball-and-socket (***) or are ball-and-socket adjacent (*). The V_H list includes all residues in the elbow region.

Supplementary Table 9. DSC thermal denaturation of the DH270 bnAb lineage mutants.

	**DSC**		DSC Model Fits		
**DH270 mAb**	**T_{onset} (°C)**	**T_m (°C)**	$T_{m,1}$ (°C)	$T_{m,2}$ (°C)	$T_{m,3}$ (°C)
UCA	58.9 ± 0.2	**85.0 ± 0.1**	65.00 ± 0.05	81.68 ± 0.05	*85.03 ± 0.01*
UCA-V11M	56.6 ± 0.7	**84.6 ± 0.1**	64.11 ± 0.04	81.66 ± 0.01	*84.75 ± 0.02*
I2	60.4 ± 0.4	**72.3 ± 0.1**	66.1 ± 0.3	*71.91 ± 0.01*	81.1 ± 0.3
I2-M11V	58.6 ± 0.3	**72.50 ± 0.02**	65.7 ± 0.2	*72.05 ± 0.03*	78.6 ± 0.7
DH270.6	59.3 ± 0.3	**64.34 ± 0.01**	63.27 ± 0.04	*64.26 ± 0.04*	78.2 ± 0.2
DH270.6-M11V	59.4 ± 0.4	**65.08 ± 0.02**	63.9 ± 0.2	*64.99 ± 0.01*	77.7 ± 0.1
DH270.6-N13K	59.0 ± 0.6	**65.44 ± 0.01**	63.7 ± 0.3	*65.33 ± 0.02*	77.3 ± 0.3
DH270.6-M11V/N13K	59.7 ± 0.6	**66.48 ± 0.02**	64.5 ± 0.3	*66.5 ± 0.1*	77.0 ± 0.1
DH270.2	53.7 ± 0.1	**59.25 ± 0.02**	*58.96 ± 0.01*	71.3 ± 0.2	81.0 ± 0.4
DH270.2-L11V	52.8 ± 0.1	**57.8 ± 0.1**	*57.57 ± 0.01*	72.7 ± 0.1	82.8 ± 0.2
DH270.2-E13K	54.8 ± 0.1	**60.6 ± 0.1**	*60.33 ± 0.04*	72.2 ± 0.5	82.6 ± 0.5
DH270.2-L11V/E13K	53.5 ± 0.1	**58.9 ± 0.1**	*58.71 ± 0.01*	72.69 ± 0.01	82.8 ± 0.1

Antibody thermal denaturation profiles were obtained and analyzed by DSC as described in Methods. The DSC raw data was best fit with three Gaussian transition models ($T_{m,1\text{-}3}$). The largest amplitude model (indicated in italics) corresponds to the denaturation of the Fab domain and is in good agreement with the observed primary T_m. Data are shown as the mean and standard deviation from a minimum of two replicate measurements.

Supplementary Table 10. Neutralization of the DH270.6 mutants.

Pseudovirus	Clade	V1 length	DH270.6	DH270.6 M11V	DH270.6 N13K	DH270.6 M11V/N13K
CH848.d4949.10.17	C	17	0.03	0.05	0.05	0.05
CH848.d836.10.31	C	17	<0.02	<0.02	<0.02	<0.02
CH848.d358.80.06	C	24	0.06	0.03	0.03	<0.02
CH848.d1432.5.41	C	30	0.4	0.6	0.6	0.6
CH848.d526.25.02	C	34	1.9	3.2	2.7	2.4
ZM55F.PB28a	C	13	0.03	<0.02	<0.02	<0.02
CAAN5342.A2	B	24	<0.02	<0.02	<0.02	<0.02
ZM106F.PB9	C	25	<0.02	<0.02	<0.02	<0.02
YU2	B	26	0.07	0.06	0.06	0.06
TRJ04551.58	B	34	0.1	0.09	0.08	0.08
DJ263.8	AG	36	0.04	0.04	0.04	0.06

IC_{50} (μg/mL)
<0.1
0.1-0.5
0.5-1
1-10
10-25
25-50
>50

Antibody neutralization was measured by TZM-bl cell-based assay using a select panel of geographically and genetically diverse Env-pseudoviruses. All values are IC_{50} in units of μg/mL.

Supplementary References

1. Lesk, A.M. & Chothia, C. Elbow motion in the immunoglobulins involves a molecular ball-and-socket joint. *Nature* **335**, 188 (1988).
2. Stanfield, R.L., Zemla, A., Wilson, I.A. & Rupp, B. Antibody Elbow Angles are Influenced by their Light Chain Class. *Journal of Molecular Biology* **357**, 1566-1574 (2006).
3. Dunbar, J., Fuchs, A., Shi, J. & Deane, C.M. ABangle: characterising the VH–VL orientation in antibodies. **26**, 611-620 (2013).
4. Fera, D. et al. Affinity maturation in an HIV broadly neutralizing B-cell lineage through reorientation of variable domains. *Proceedings of the National Academy of Sciences* **111**, 10275-10280 (2014).
5. Williams, W.B. et al. Initiation of HIV neutralizing B cell lineages with sequential envelope immunizations. *Nature Communications* **8**(2017).
6. Liao, H.X. et al. Co-evolution of a broadly neutralizing HIV-1 antibody and founder virus. *Nature* **496**, 469-76 (2013).
7. Bonsignori, M. et al. Antibody-virus co-evolution in HIV infection: paths for HIV vaccine development. *Immunol Rev* **275**, 145-160 (2017).
