# Supplementary material for: Selection of immunoglobulin elbow region mutations impacts interdomain conformational flexibility in HIV-1 broadly neutralizing antibodies
Source: Nat Commun. 2019 Feb 8;10:654. doi: 10.1038/s41467-019-08415-7 (PMC6368608; doi:10.1038/s41467-019-08415-7)
Supplement: Supplementary file 2 — Description of Additional Supplementary Files [file 41467_2019_8415_MOESM2_ESM.pdf]

Description of Additional Supplementary Files

File Name: Supplementary Movie 1
Description: Representative CH103 UCA and CH103 P14S/S30G Simulations. Top two panels depict CH1/CL fixed Fabs demonstrating the shifts in elbow angles. Bottom panels depict elbow region residues (sticks) with the ball and socket residue surfaces (magenta) and position 14 surface (yellow) highlighted.

File Name: Supplementary Movie 2
Description: Representative DH270 UCA and DH270 I2 Simulations. Top two panels depict CH1/CL fixed Fabs demonstrating the shifts in elbow angles. Bottom panels depict elbow region residues (sticks) with the ball and socket residue surfaces (magenta) and position 11 mutation site surface (yellow) highlighted.

File Name: Supplementary Movie 3
Description: Representative CH58 UCA and CH58 Simulations. Top two panels depict CH1/CL fixed Fabs demonstrating the shifts in elbow angles. Bottom panels depict elbow region residues (sticks) with the ball and socket residue surfaces (magenta) highlighted.
